# *Vibrio* sp. dhg as a platform for the biorefinery of brown macroalgae

**DOI:** 10.1038/s41467-019-10371-1

**Published:** 2019-06-06

**Authors:** Hyun Gyu Lim, Dong Hun Kwak, Sungwoo Park, Sunghwa Woo, Jae-Seong Yang, Chae Won Kang, Beomhee Kim, Myung Hyun Noh, Sang Woo Seo, Gyoo Yeol Jung

**Affiliations:** 1 Department of Chemical Engineering, Pohang University of Science and Technology, 77 Cheongam-Ro, Nam-Gu, Pohang, Gyeongbuk 37673 Korea; 2 School of Interdisciplinary Bioscience and Bioengineering, Pohang University of Science and Technology, 77 Cheongam-Ro, Nam-Gu, Pohang, Gyeongbuk 37673 Korea; 3 School of Chemical and Biological Engineering, Institute of Chemical Process, Seoul National University, 1 Gwanak-Ro, Gwanak-Gu, Seoul, 08826 Korea

**Keywords:** Microbiology techniques, Metabolic engineering, Metabolic engineering

## Abstract

Although brown macroalgae holds potential as an alternative feedstock, its utilization by conventional microbial platforms has been limited due to the inability to metabolize one of the principal sugars, alginate. Here, we isolate *Vibrio* sp. dhg, a fast-growing bacterium that can efficiently assimilate alginate. Based on systematic characterization of the genomic information of *Vibrio* sp. dhg, we establish a genetic toolbox for its engineering. We also demonstrate its ability to rapidly produce ethanol, 2,3-butanediol, and lycopene from brown macroalgae sugar mixture with high productivities and yields. Collectively, *Vibrio* sp. dhg can be used as a platform for the efficient conversion of brown macroalgae sugars into diverse value-added biochemicals.

## Abstract (web summary)

Brown macroalgae is a good candidate feedstock for biorefinery, but the major carbohydrate alginate cannot be digested by current industrial microbes. Here, the authors isolate *Vibrio* sp. dhg and engineer it to produce value-added biochemicals from alginate using newly developed genetic tools.

## Introduction

The global demand for bioproducts is increasing at a striking rate, with its market share expected to reach 22% of the chemical industry by 2025^1^. To meet such a vast demand, stable supplementation and conversion of feedstocks have been critical over the past years. Although starch crops have been widely used up until now, many concerns exist regarding the consumption of food resources and limited cultivation capabilities. In this respect, brown macroalgae have been suggested as an alternative feedstock. Brown macroalgae are hugely abundant and have a high carbohydrate content (35–60% of dry weight)^2–4^. They can grow much faster than lignocellulosic biomasses and only require sunlight and seawater^5^.

The most prominent sugars in brown macroalgae are alginate (a copolymer of α-L-guluronate and β-D-mannuronate) and mannitol. While a conventional microbial platform (e.g., *Escherichia coli*) can easily metabolize mannitol, its ability to assimilate alginate is hindered by the fact that it lacks certain related genes; it is known that alginate metabolism requires about 10–20 genes that encode transporters, lyases, and metabolic enzymes^6,7^. Although recent studies have demonstrated that *E. coli* can be engineered to utilize alginate with introduction a huge gene cluster for alginate utilization from naturally-occurring alginate-metabolizing microorganism^6,8^, their growth rates and metabolic activities are still too low for industrial applications, likely due to the unoptimized expression of multiple xenogeneic genes.

As both the growth rate and the metabolic activity of host microorganisms greatly affect the performance of bioprocesses, it is crucial to exploit an efficient host with high rates of both these factors in order to obtain a high productivity. Therefore, microorganisms that are naturally capable of using alginate should be considered as the preferred microbial platform for brown macroalgae feedstocks. Due to natural optimization throughout evolutionary history, such microorganisms likely have a superior capacity to metabolize alginate compared with engineered versions of conventional microbes. Thus, these naturally occurring microorganisms would be more suitable as a microbial platform for producing diverse value-added biochemicals from the sugars of brown macroalgae.

In this study, we isolate a fast-growing microorganism capable of utilizing alginate efficiently. We name this microorganism as *Vibrio* sp. dhg and characterize it systematically to develop a genetic engineering toolbox. By exploitation as a microbial platform, we demonstrate diverse value-added biochemical production from brown macroalgae sugars with high productivities and yields. From these results, we propose *Vibrio* sp. dhg as a platform for the biorefinery of brown macroalgae.

## Results

### Isolation of an alginate-utilizing microorganism

**Fig. 1:**
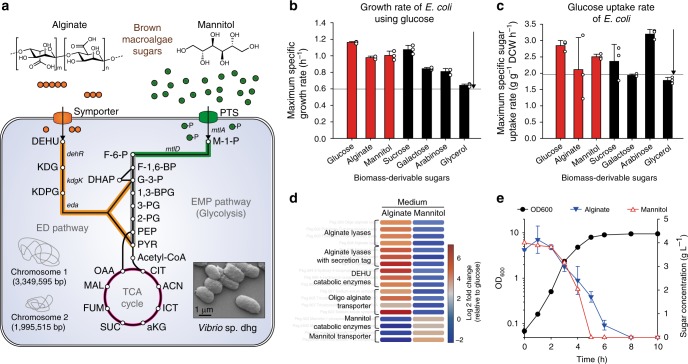
*Vibrio* sp. dhg as a microbial platform for brown macroalgae biorefinery. **a** Schematic alginate and mannitol assimilation pathways in *Vibrio* sp. dhg. Depolymerized oligo-alginate is transported into the cytosol and digested into 4-deoxy-L-erythro-5-hexoseulose uronate (DEHU). DEHU is further converted to glyceraldehyde 3-phosphate (G-3-P) and pyruvate (PYR). Mannitol is transported into the cytosol by the PTS system and is converted to fructose-6-phosphate (F-6-P). The inset is a scanning electron microscopy (SEM) image of *Vibrio* sp. dhg with a scale bar indicating 1 μm. The other abbreviations are as follows: ED pathway Entner–Doudoroff pathway, EMP pathway Embden–Meyerhof–Parnas pathway, KDG 2-keto-3-deoxygluconate, KDPG 2-keto-3-deoxy-6-phosphogluconate, M-1-P mannitol-1-phosphate, F-1,6-BP fructose 1,6-bisphosphatase, DHAP dihydroxyacetone phosphate, 1,3-BPG 1,3-bisphosphoglycerate, 3-PG 3-phosphoglycerate, 2-PG 2-phosphoglycerate, PEP phosphoenolpyruvate, CIT citrate ACN *cis*-aconitate, ICT isocitrate, aKG a-ketoglutarate, SUC succinate, FUM fumarate, MAL malate, and OAA oxaloacetate. **b** Maximum specific growth rates (h^−1^) and **c** maximum sugar uptake rates (g g^−1^ dry cell weight (DCW) h^−1^) of *Vibrio* sp. dhg in minimal medium supplemented with 4 g L^−1^ of diverse carbon sources at 30 °C. The two horizontal lines indicate growth rate (0.60 h^−1^) and sugar uptake rate (1.96 g g^−1^ DCW h^−1^) of *E. coli* in glucose minimal medium at 37 °C. **d** Fold changes of gene expression during alginate and mannitol assimilation as compared with during glucose assimilation, based on RNA-Seq results. **e** Simultaneous assimilation of alginate and mannitol by *Vibrio* sp. dhg. Closed black circles, OD_600_; closed inverted triangles, alginate; open red triangles, mannitol. Error bar indicates the standard deviations of three independent cultures (*n* = 3). White dot indicates actual data point. Source data of Figs. 1b, 1c, and 1d are provided as a Source Data file

To isolate alginate-utilizing microorganism, seaweed sludge was collected and inoculated in minimal medium supplemented with alginate as a sole carbon source (Supplementary Note 1). After a few rounds of sub-culturing at 30 °C, a rod-shaped microorganism showing a rapid growth (maximum specific growth rate (*μ*) = 0.98 h^−1^) was successfully isolated (Fig. 1a). This microorganism is able to use not only alginate but also other biomass-derivable sugars (e.g., glucose, mannitol, sucrose, galactose, arabinose, and glycerol). Notably, with most sugars, the rates of growth and sugar uptake at 30 °C were substantially higher or at least comparable with those of *E. coli* grown with glucose at 37 °C (Fig. 1b, c).

In addition, this microorganism can grow even in a high concentration of salt (100 g L^−1^ of sodium chloride) while other industrial hosts (*E. coli*, *Corynebacterium glutamicum*, *Saccharomyces cerevisiae*, Supplementary Data 1, and Supplementary Fig. 1a) showed severely reduced growth rates. Tolerance to other representative bioproducts (ethanol, 2,3-butanediol (2,3-BDO), and lactate) was also investigated (Supplementary Fig. 1b–d). In the case of ethanol, this microorganism was similar to *C. glutamicum* and maintained its growth with 50 g L^−1^ of ethanol in the medium. For 2,3-BDO, it was similar to *E. coli* but more sensitive than *C. glutamicum* and *S. cerevisiae*; the growth was inhibited by 50 g L^−1^ of 2,3-BDO. Finally, with lactate, its tolerance was slightly less than the tolerance of *S. cerevisiae* which showed the best growth in the presence of 50 g L^−1^ of lactate. Overall, although its tolerance was not much superior to either *C. glutamicum* or *S. cerevisiae*, it was comparable to *E. coli*, suggesting this microorganism can be used as a host for biochemical production.

To identify this microorganism, we analyzed its 16S rDNA sequence using the universal primers (Supplementary Data 2) and found that it belongs to the family *Vibrionaceae*. In particular, it was highly similar to *Vibrio natriegens* ATCC14048 (99% identity, Supplementary Data 3), a gram-negative bacterium recently suggested to be a promising host for molecular biotechnology due to its rapid growth rate (doubling time < 10 min in rich medium)^9,10^. Upon sequencing the genome of our isolated microorganism (Supplementary Fig. 2, Supplementary Tables 1 and 2), three circular contigs representing a large chromosome, a small chromosome, and a plasmid were obtained. Although several *Vibrio* species are known to use alginate (e.g., *V. alginolyticus* and *V. splendidus* 12B01), its growth rate is much higher than reported values of the known microorganisms (0.2–0.8 h^−1^)^7^. Moreover, the genome context and phenotypic characteristics of our microorganism were closer to those of *V. natriegens* ATCC14048 (Supplementary Figs. 3 and 4). However, detailed physiological comparison (Supplementary Table 3) revealed that there is a difference between two microorganisms, probably due to a variation in more than 5% of their genomes. Particularly, *V. natriegens* does not possess the machinery needed to metabolize alginate. Therefore, we named our strain *Vibrio* sp. dhg and further investigated its potential as a platform for biorefinery from brown macroalgae.

Genome annotation revealed that many of the genes responsible for alginate assimilation in *Vibrio* sp. dhg were located in a 42-kb cluster within its small chromosome (chromosome 2, Supplementary Data 4). Sequence comparison with a well-studied gene cluster of *V. splendidus*^6^ indicated that our strain also assimilates alginate via a multi-step process (Fig. 1a). Specifically, endolytic alginate lyases are secreted to depolymerize alginate into oligo-alginates^11^. The oligo-alginates are then transported into the cytosol through a transporter. Subsequently, alginate monomers (4-deoxy-L-erythro-5-hexoseulose uronate, DEHU) are produced by the action of exolytic oligo-alginate lyases. Finally, via a partial Entner–Doudoroff pathway, DEHU reductase (DehR), 2-keto-3-deoxygluconate kinase (KdgK) and 2-keto-3-deoxyphosphogluconate aldolase (Eda) convert DEHU into glyceraldehyde-3-phosphate and pyruvate, two metabolites that then enter glycolysis. On the other hand, the large chromosome contains two copies of the mannitol operon (chromosome 1). Mannitol is initially transported into the cell by a mannitol-specific phosphotransferase system (PTS, MtlA) and then converted into fructose-6-phosphate—an intermediate of glycolysis—by mannitol-1-phosphate dehydrogenase (MtlD).

To confirm that the aforementioned genes are critical for alginate and mannitol assimilation, we analyzed their expression levels via RNA sequencing. As expected, these genes were highly induced by the addition of the cognate sugar (up to a 100-fold increase compared with the glucose condition) (Fig. 1d). Additionally, we observed that *Vibrio* sp. dhg could simultaneously consume alginate and mannitol (Fig. 1e). As these two sugars have different oxidation states, this property is highly advantageous for biochemical production (e.g., ethanol) in an oxygen-limited environment (see Supplementary Note 2)^12,13^. Thus, *Vibrio* sp. dhg is highly suited to using an alginate-mannitol mixture.

### Gene expression control in *Vibrio* sp. dhg

To harness *Vibrio* sp. dhg as a microbial platform, genetic engineering tools for the construction and optimization of (heterologous) metabolic pathways must first be readily available. As an elemental approach, we introduced several plasmids with different origins of replication (pMB1, RSF1030, p15A, and CloDF13) into *Vibrio* sp. dhg strain. Using a transformation protocol originally established for *V. natriegens*^10^, we observed that all plasmids except CloDF13 could stably exist (Supplementary Fig. 5). Furthermore, more than two different plasmids could be maintained under the presence of multiple antibiotics.

**Fig. 2:**
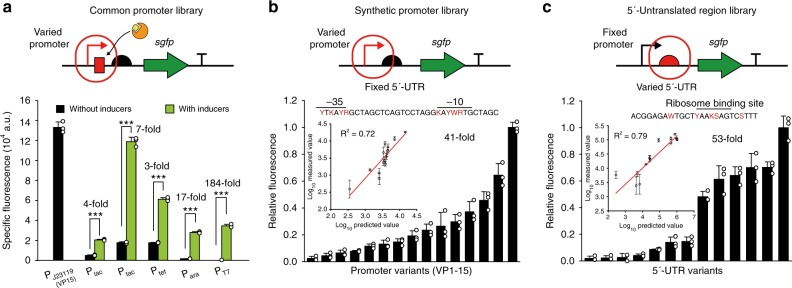
Precise control of gene expression in *Vibrio* sp. dhg. **a** Evaluation of a constitutive promoter (P_J23119_, equivalent to P_VP15_) and various inducible promoters (P_lac_, P_tac_, P_tet_, P_ara_, and P_T7_) in terms of *sgfp* expression. For inducible expression, either 1 mM IPTG, 100 μg mL^−1^ aTc, or 10 g L^−1^ arabinose was added. ***p < 0.001 (two-sided *t*-test). **b** Relative fluorescence values for *sgfp* expression under the control of various synthetic promoters. Each fluorescence value was normalized by the highest value. The inset graph indicates a positive correlation (*R*^2^ = 0.72) between the predicted promoter strength and the measured fluorescence values. **c** Relative fluorescence values for *sgfp* expression in function of various 5′-UTRs. Each fluorescence value was normalized by the highest value. The inset graph indicates a high correlation (*R*^2^ = 0.79) between the predicted folding energies (ΔG_UTR_) and the measured fluorescence values. Values were adjusted for background fluorescence (fluorescence of the VDHG001 strain). Error bar indicates the standard deviations of three independent cultures (*n* = 3). White dot indicates actual data point. Source data of Figs. 2a, 2b, and 2c are provided as a Source Data file

Next, we investigated the features of transcription that drive gene expression in *Vibrio* sp. dhg. Upon comparing the amino acid sequence of its housekeeping sigma factor (σ_70_) with that of *E. coli*, we noticed that the major DNA-binding domains (region 2.4 and 4.2) were 100% identical (Supplementary Fig. 6). Furthermore, the promoter regions of ribosomal genes, which are highly expressed in *Vibrio* sp. dhg, also have similar sequences (TTGANN and TATAAT) to the bacterial −35 and −10 boxes (TTGACA and TATAAT, respectively) (Supplementary Fig. 7). These findings suggested that the common promoters used for *E. coli* would also be compatible with *Vibrio* sp. dhg. To test this idea, we prepared plasmids encoding the *sgfp* (super-folding green fluorescence protein) gene under the control of the conventional promoters P_J23119_, P_lac_, P_tac_, P_tet_, and P_ara_. These plasmids also carry cognate repressor expression cassettes (*lacI* and *araC* cassettes from *E. coli*, and a synthetically designed *tetR* cassette) for inducible expression. As expected, we were able to observe successful expression of the *sgfp* gene in *Vibrio* sp. dhg when transcribed by the endogenous RNA polymerase (Fig. 2a). In addition, expression of the T7 RNA polymerase gene (under the control of the P_lac_ promoter) also resulted in the expression of *sgfp* when placed under the control of the P_T7_ promoter. Furthermore, all inducible promoters showed strong activation (up to a maximum of 184-fold) upon the addition of cognate inducers (isopropyl β-D-1-thiogalactopyranoside (IPTG), anhydrotetracycline (aTc) or arabinose).

To achieve precise control of gene expression at the transcriptional level, we constructed a synthetic promoter library. This library was prepared by modifying the −35 and −10 boxes of the P_J23119_ promoter with degenerate sequences (i.e., YTKAYR and KAYWRT, respectively) to cover the Anderson promoter library^14^. By altering the promoter sequence, we successfully obtained 15 different promoters (named promoters VP1 to 15) of varied strengths, with a maximum fold difference of 41 (Fig. 2b, Supplementary Table 4). Based on our data, we were able to construct a predictive model that can estimate promoter strengths (Supplementary Table 5) with a strong predictive power (*R*^2^ = 0.72). In agreement with the observed strengths, our model predicted promoter VP15 to be the strongest. Thus, our model could be useful in the development of other synthetic promoters and be further improved by iterative-learning.

We also investigated gene expression control in *Vibrio* sp. dhg at the translational level. Recently, prediction of translation efficiency in bacteria became possible thanks to a biophysical model of the translation initiation process^15,16^. This model, which has been thoroughly tested in *E. coli*, calculates the thermodynamic free energy change of the 5′-untranslated region (ΔG_UTR_). Although the 16S rDNA of *Vibrio* sp. dhg (Supplementary Data 3) has a high similarity (90%) and the same anti-Shine-Dalgarno sequence (ACCTCCTTA) to the 16 S rDNA of *E. coli*, the biophysical model still requires validation in *Vibrio* sp. dhg. Thus, to confirm the predictable nature of translational efficiency in *Vibrio* sp. dhg, we generated a 5′-UTR library covering a broad range of *sgfp* expression levels using UTR Library Designer^16^ (Supplementary Table 6). In total, we analyzed 13 5′-UTR variants and found that gene expression was successfully varied. More specifically, alteration of the ribosome binding site and the folding features^16^ enabled us to generate variants with a difference in expression of up to 53-fold (Fig. 2c). More importantly, these measured values were highly correlated (*R*^2^ = 0.79) with the predicted values, thereby indicating a strong predictive power for this model in *Vibrio* sp. dhg. Taken together, both transcription and translation can be controlled in a predictable manner in *Vibrio* sp. dhg, a property that is highly useful for the construction of efficient metabolic pathways. Furthermore, many plasmid-based production systems or genetic circuits, which have been previously developed for *E. coli*, can be directly employed in *Vibrio* sp. dhg without any major modifications.

### Genome editing in *Vibrio* sp. dhg

As genome editing is necessary to develop an efficient cell factory, the possibility to engineer the genome of *Vibrio* sp. dhg was explored. To engineer its genome, the SXT recombination system naturally found in *Vibrionaceae* was chosen. It has been reported that the expression of *exo* (a gene encoding alkaline exonuclease) and *beta* (a gene encoding single-strand annealing protein) permits recombination in *V. natriegens* and *E. coli*^17,18^. For its genome engineering, endogenous *exo* and *beta* in *Vibrio* sp. dhg, were expressed under the control of the P_tac_ promoter and a synthetic 5′-UTR (Supplementary Table 7). Additionally, *gamma* (a gene encoding nuclease inhibitor) from lambda phage was expressed to facilitate recombination^17^. As a template for allelic exchange, we introduced a chloramphenicol resistance (*cat*) cassette flanked with two identical FRT sequences^19^ and homology arms (1–3 kb) of the target locus^10^. After electroporation, we confirmed the engineered locus by checking for colonies that could grow on a chloramphenicol agar plate. Indeed, we found colonies with target gene successfully replaced by the *cat* gene (Supplementary Fig. 8a). Subsequently, the integrated *cat* gene was easily removed by expressing an FRT-specific flippase from *S. cerevisiae*^19^.

### Biochemical production from alginate and mannitol mixture

**Fig. 3:**
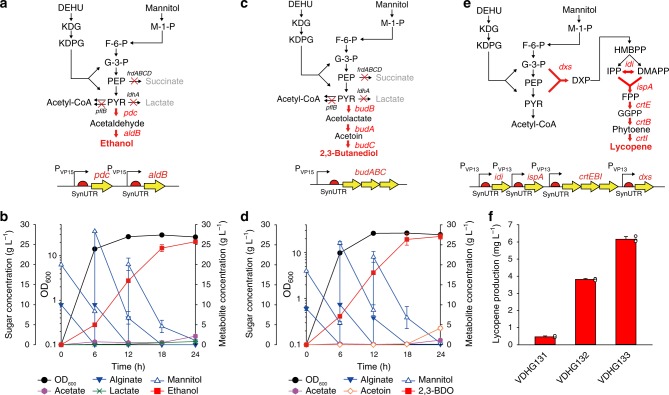
Biochemical production using engineered *Vibrio* sp. dhg strains. **a** Ethanol production pathway. For ethanol production, *pdc* (pyruvate decarboxylase) and *aldB* (aldehyde dehydrogenase) from *Z. mobilis* were expressed under the P_VP15_ promoter and a synthetic 5′-UTR. To improve yield, *ldhA*, *frdABCD*, and *pflB* were removed from the chromosome. **b** Fermentation profile of the VDHG411 strain during 24 h. Closed black circle, OD_600_; closed inverted blue triangle, alginate; open blue triangle, mannitol; closed purple hexagon, acetate; green cross, lactate; closed red square, ethanol. **c** 2,3-BDO production pathway. The *budABC* operon from *E. aerogenes* was expressed under the P _VP15_ promoter and a synthetic 5′-UTR. **d** Fermentation profile of the VDHG421 strain during 24 h. Closed black circle, OD_600_; closed inverted blue triangle, alginate; open blue triangle, mannitol; closed purple hexagon, acetate; open orange diamond, acetoin; closed red square, 2,3-BDO. **e** Lycopene production pathway. For lycopene production, the *crtEBI* operon (encoding GGPP synthase, phytoene synthase, and phytoene desaturase) from *L. purpurea* was expressed under the P_VP13_ promoter and a synthetic 5′-UTR. To improve the titer, *dxs* (DXS synthase), *idi* (IPP isomerase) and *ispA* (FPP syntase) from *E. coli* W3110 were additionally expressed. **f** Lycopene production by engineered *Vibrio* sp. dhg strains (VDHG131, VDHG132, and VDHG133) after 9 h. Abbreviations: DXP deoxyxylulose 5-phosphate, HMBPP 4-hydroxy-3-methylbut-2-enyl pyrophosphate, IPP isopentenyl pyrophosphate, DMAPP dimethylallyl pyrophosphate, FPP farnesyl diphosphate, GGPP geranylgeranyl pyrophosphate. Some reactions of glycolysis have been omitted for simplicity. Error bar indicates the standard deviations of three independent cultures (*n* = 3). White dot indicates actual data point

After establishing a genetic toolbox for *Vibrio* sp. dhg, we attempted to produce value-added biochemicals using engineered *Vibrio* sp. strains. Although sugar composition of brown macroalgae is known to be influenced by geographical and seasonal changes^20^, a mixture of alginate and mannitol in a 1:2 ratio was chosen to prepare a mimetic sugar medium to fully evaluate its performance. At first, production of ethanol, one of biofuels, was attempted in a microaerobic condition (Fig. 3a). Since the wildtype strain possesses both an aldehyde dehydrogenase and an alcohol dehydrogenase, ethanol can be produced by cultivating the wildtype strain via reduction of acetyl-CoA. However, only 1.6 g L^−1^ of ethanol was produced from 30 g L^−1^ of mixed sugars (Supplementary Fig. 9). In addition, other by-products such as acetate, lactate, and succinate were produced in considerable amounts (a total of 4.7 g L^−1^). To produce more ethanol, the pyruvate decarboxylase (pdc) and aldehyde dehydrogenase (*aldB*) genes of *Zymomonas mobils*^6^ (Fig. 3a) were expressed. To obtain constitutive expression at the maximum possible level, we fused each gene to a synthetic 5′-UTR (Supplementary Table 7) and placed them under the control of the strongest synthetic promoter, VP15. The resulting strain showed dramatically improved ethanol production (7.7 g L^−1^, or a 4.8-fold increase) and lower levels of accumulated by-products (a total of 2.3 g L^−1^). To further minimize by-product formation, we deleted the endogenous *ldhA* (lactate dehydrogenase), *frdABCD* (fumarate reductase) and *pflB* (pyruvate-formate lyase) genes, creating the VDHG411 strain (Supplementary Fig. 8b). This deletion successfully minimized the loss of carbon (a total of only 1.1 g L^−1^ of by-products) while slightly improving ethanol production (8.4 g L^−1^, or a 10% increase). Upon providing a total of 80 g L^−1^ of mixed sugars with a 1:2 ratio by fed-batch fermentation, VDHG411 strain produced 25.7 g L^−1^ (3.3% v/v) of ethanol in 24 h (Fig. 3b). Surprisingly, this 1.1 g L^−1^ h^−1^ average ethanol productivity (maximum 1.8 g L^−1^ h^−1^) was dramatically higher than that of other microbial platforms for alginate utilization (Supplementary Table 8). Furthermore, the yield (64% of the theoretical maximum) of our VDHG411 strain was comparable to the yield of other alginate-consuming microbial platforms.

We next attempted to produce 2,3-BDO, a chemical that can be used industrially as an antifreeze reagent or solvent^21^ (Fig. 3c). Although the production of 2,3-BDO from the sugars of brown macroalgae has previously been attempted^21^, this was carried out using *E. coli*, which is only able to slowly assimilate mannitol. In contrast, alginate has not yet been considered as a carbon source in such a bioprocess. Thus, to produce 2,3-BDO using our alginate-assimilating *Vibrio* sp. dhg, we expressed the *budABC* operon (encoding for acetolactate decarboxylase, acetolactate synthase, and acetoin reductase) from *Enterobacter aerogenes*^21^ under the P_VP15_ promoter and a synthetic 5′-UTR (Supplementary Table 7). The heterologous expression of this operon enabled the production of 6.1 g L^−1^ of 2,3-BDO from 30 g L^−1^ of mixed sugars in 9 h (Supplementary Fig. 10). Moreover, by deleting the competing pathways (i.e., Δ*ldhA*, Δ*frdABCD*, and Δ*pflB*), we were able to further improve production 1.5-fold (9.4 g L^−1^, the VDHG421 strain). By fed-batch fermentation, this strain was able to produce 27.1 g L^−1^ of 2,3-BDO (31.3 g L^−1^ including acetoin) from 80 g L^−1^ of sugars in 24 h (Fig. 3d). When compared with the *E. coli* platform of the previously mentioned study^21^, *Vibrio* sp. dhg showed both superior productivity (1.3 g of 2,3-BDO and acetoin L^−1^ h^−1^) and yield (0.40 g product g^−1^ sugar or 81% of the theoretical maximum), along with an efficient co-utilization of alginate and mannitol.

We lastly demonstrated the ability of our *Vibrio* sp. dhg platform to produce lycopene, which is a C-40 phytochemical used as a nutraceutical antioxidant^22,23^. We first prepared the required plasmids (Supplementary Data 1) by re-organizing previously reported plasmids^23^ that express *crtEBI* (geranylgeranyl pyrophosphate (GGPP) synthase, phytoene synthase and phytoene desaturase) from *Lamprocystis purpurea*, and *dxs* (1-deoxy-D-xylulose-5-phosphate (DXP) synthase), *idi* (isopentenyl diphosphate (IPP) isomerase) and *ispA* (farnesyl diphosphate (FPP) synthase) from *E. coli* (Fig. 3e). These genes were expressed under the control of the P_VP13_ promoter and synthetic 5′-UTRs^23^ (Supplementary Table 7). When we supplemented strain VDHG131—the strain expressing only the essential lycopene genes (*crtEBI*) – with 10 g L^−1^ of sugar mixture, 0.47 mg L^−1^ of lycopene was produced in 9 h (Fig. 3f). Additional expression of *idi* and *ispA* (strain VDHG132) or *idi*, *ispA*, and *dxs* (strain VDHG133) successfully increased lycopene production (3.8 mg L^−1^ and 6.2 mg L^−1^ for the two strains, respectively). Compared with a similarly constructed *E. coli* strain (~4 mg L^−1^ during 24 h)^23^, the use of *Vibrio* sp. dhg enabled a much faster production of lycopene (more than a fourfold increase in productivity). As slow growth and low accumulation of biomasses are known hurdles for efficient production of lycopene, the fast-growing *Vibrio* sp. dhg could be an ideal host. Importantly, additional engineering—such as precursor balancing^23^—using our developed toolbox could enhance lycopene production even further.

### Brown macroalgae fermentation for ethanol production

**Fig. 4:**
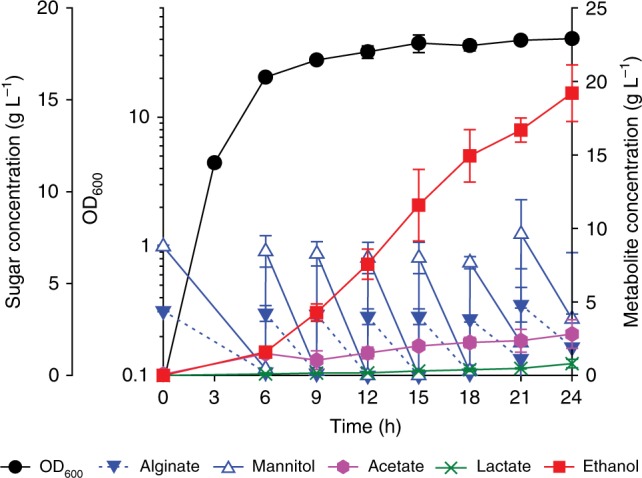
Brown macroalgae fermentation for ethanol production. A brown macroalgae fermentation profile of the VDHG411 strain using a 5 L bioreactor during 24 h. Residual alginate concentration was roughly estimated based on mannitol concentration using the measured kelp composition due to difficulty in its quantification using concentrated kelp medium. OD_600_ values after 6 h may not represent the exact amount of biomass due to turbidity from the Kelp powder. Closed black circle, OD_600_; closed inverted blue triangle, alginate; open blue triangle, mannitol; closed purple hexagon, acetate; green cross, lactate; closed red square, ethanol. Error bar indicates the standard deviations of three independent cultures (*n* = 3)

Finally, we tested ethanol production using brown macroalgae as feedstock (Fig. 4). The sugar content of used Kelp powder was quantified to 0.16 g alginate and 0.3 g mannitol per g powder (see “Methods”), consistent with the composition of the mimetic sugar mixture. Therefore, the Kelp powder can be directly used as a feedstock. When the VDHG411 strain was cultivated in a bioreactor containing a 1 L medium, total 120 g L^−1^ of the Kelp powder was provided during 24 h. Subsequently, even without any enzymatic pre-treatment or hydrolysis, 19.2 g L^−1^ of ethanol (2.4% v/v) was successfully produced with a similar yield (63% of theoretical maximum) compared to the yield from mimetic sugar fermentation. Although the productivity (0.8 g L^−1^ h^−1^) was reduced, suggesting a requirement of further optimization including genetic engineering and parameter study, the obtained result clearly indicates that *Vibrio* sp. dhg is also capable to produce a biochemical directly from brown macroalgae.

## Discussion

The ultimate goal of the biorefinery process is the efficient conversion of abundant carbon sources into value-added biochemicals. While the use of brown macroalgae as a feedstock has been limited due to the difficulties of alginate assimilation, the use of *Vibrio* sp. dhg strain as a microbial platform could overcome this issue. Moreover, its outstanding growth rate and metabolic activity over conventional platforms allow envisioning accelerated biochemical production from brown macroalgae.

One risk in exploiting environmental isolates as a host is their potential virulence. Our genome analysis revealed that *Vibrio* sp. dhg strain does not have major toxins found from pathogenic *Vibrio* strains^10^ (see “Methods”). However, one barely expressed open reading frame (fragments per kilobase per million (FPKM) < 1 based on RNA-seq analysis) showed a marginal identity (24%, Supplementary Fig. 11a and b) when compared with its amino acid sequence with an auxiliary toxin (Zot, zona occludens toxin). Although it has a low sequence identity (less than 30%^24^) and *Vibrio* sp. dhg strain does not have two major toxins (CtxA and CtxB) concurrently found with Zot^25^, we tested that the potential toxin can be deleted to minimize the risk. We confirmed that this gene can be deleted and does not affect the growth of the strain (Supplementary Fig. 11c and d).

For the realization of the brown macroalgae biorefinery, there could still be many hurdles. As mentioned, one challenge is a potential fluctuation of bioprocess efficiency due to seasonal and geographical differences in biomass sugar composition. Indeed, we observed that the ratio of alginate and mannitol, which have different oxidation states, hugely affected ethanol production (up to 4.5-fold) (Supplementary Fig. 12 and Supplementary Note 2). To address this issue, microorganisms should be engineered for dynamic response to intracellular redox state for robust biochemical production with advantages of recent advances in synthetic biology^26–29^.

Further studies aimed at unveiling the characteristics (e.g., rapid growth, halophilicity) and improving its tolerance^30,31^ of *Vibrio* sp. dhg would be valuable for maximizing its potential as a microbial platform^32^. Additionally, the exploitation of fast-growing microorganisms as hosts can also expedite evolutionary processes that lead to the development of improved proteins or microorganisms themselves. The strain development repertoire that we demonstrate here, could also be applied to the construction of other microbial platforms that utilize non-traditional carbon sources^33,34^. By combining all these efforts, we will likely be able to achieve the ultimate goal of the biorefinery in the near future.

## Methods

### Chemical reagents and oligonucleotides

Primers were synthesized by Cosmogenetech (Seoul, Korea) and are listed in Supplementary Data 2. Plasmid and genomic DNA were prepared using the GeneAll^R^ Exprep^TM^ Plasmid SV kit and the Exgene^TM^ Cell SV kit (GeneAll, Seoul, Korea), respectively. Procedures for plasmid cloning are explained in Supplementary Note 3. For purification of fragmented DNA, we used the Expin^TM^ Gel SV and Expin^TM^ PCR SV kits. Q5 polymerase, the NEBuilder^R^ HiFi DNA Assembly Cloning Kit, restriction enzymes, and the Quick Ligation^TM^ kit were purchased from New England Biolabs (Ipswich, MA, United States). For routine colony PCR, EmeraldAmp^®^ GT PCR Master Mix was used (Clontech, Mountain View, CA, USA). All reagents for cell cultures were purchased from BD Bioscience (Sparks, MD, USA). Chemical reagents were purchased from Sigma (St. Louis, MO, USA) unless otherwise stated. Buffer and medium compositions were described in Supplementary Note 1.

### Isolation of *Vibrio* sp. dhg strain

To isolate an alginate-metabolizing microorganism, we obtained seaweed sludge from the coastal area of Pohang, Korea. The sludge sample was inoculated into 20 mL of alginate minimal medium in a 350-mL flask. When growth was detected by a change in turbidity, the culture was transferred to the same fresh medium using a 1/100 dilution. After several sub-culture, the broth was streaked on alginate agar plate. We sequenced the 16 S rDNA of 10 colonies using the 16S_27F and 16S_1492R primers (Supplementary Data 2). All samples had an identical sequence and one of the sequenced colonies was named *Vibrio* sp. dhg.

### Strain characterization

For routine cell cultures, colonies were picked from rich medium agar plates (LBv2 medium for *Vibrio* sp. dhg, LB medium for *E. coli*, BHI medium for *C. glutamicum*, and YPD medium for *S. cerevisiae*) and inoculated in 3 mL of minimal medium (buffered medium for *Vibrio* sp. dhg, M9 medium for *E. coli*, CG medium for *C. glutamicum*, and SC medium for *S. cerevisiae*) supplemented with 4 g L^−1^ of a single sugar (glucose, mannitol, alginate, sucrose, arabinose, galactose, or glycerol). After growing overnight, the culture was refreshed by inoculating into fresh medium at an OD_600_ of 0.05–0.1. When the OD_600_ reached 1.0, the culture broth was transferred into either 350-mL flask (aerobic culture) containing 20 mL of medium or 175-mL serum bottle (anaerobic culture) containing 50 mL of medium. The cultures were conducted in a rotary shaker (Hanil Scientific, Gimpo, Korea) at 30 °C and 200 rpm (rotation per minute) unless otherwise mentioned. Especially, cells were handled in the anaerobic chamber (Coy Laboratories, Ann Arbor, MI, USA) when cells were anaerobically cultivated.

For the characterization of *Vibrio* sp. dhg in a reactor-scale, the refreshed seed was inoculated into a 5 L bioreactor (LiFlus GX, Hanil Scientific) containing the 1 L buffered medium with 10 g L^−1^ of glucose. 2 L min^−1^ of sterile air was provided and culture was stirred at 500 rpm. For an anaerobic culture, 50 mM of NaHCO_3_ was additionally supplemented and CO_2_ and N_2_ gas mixture (20:80) was provided instead of air.

To test tolerance of the microorganisms, cells were cultured in 100-μL minimal media contained in 96-well microtiter plate with orbital shaking (300 rpm). Different amounts (0, 10, 20, 50, and 100 g L^−1^) of sodium chloride, ethanol, lactate, 2,3-BDO were included in the medium. Lactate was pre-neutralized with sodium hydroxide. The growth of cells was measured using the Hidex Sense microplate reader (Hidex, Turku, Finland).

All cell cultures were conducted in biological triplicates. Initial OD_600_ for all cell culture was 0.05–0.1. An OD_600_ of 1.0 corresponds to 0.27 g DCW per L^9^. Maximum specific growth rate (μ, h^−1^) was calculated by linear regression of ln(OD_600_) and time (h) during exponential growth phase. To determine maximum specific sugar uptake rate (g g^−1^ DCW L^−1^), maximum specific growth rate was divided by biomass yield^9^.

### Scanning electron microscopy

Cells were cultured overnight in LBv2 medium and then sub-cultured in fresh medium by diluting 100-fold. Upon reaching an OD_600_ of 0.6, the cells were collected by centrifugation at 3700 × *g* for 10 min and washed three times with buffer solution (100 mM of potassium phosphate and 10 g L^−1^ of NaCl). Subsequently, cells were fixed by incubation in the same solution containing 2.5% glutaraldehyde. After 1 h incubation, the cell pellet was harvested by centrifugation. The resulting pellet was resuspended in increasing concentrations of ethanol (30, 50, 70, 80, and 100% v/v) in the same buffer for dehydration. Then, 1 mL of suspension was dropped on a slide glass and dried at room temperature to remove ethanol. The sample was then coated with Pt in the presence of argon gas using a metal sputter. Finally, the sample was imaged at a 5 kV acceleration voltage on a Jeol scanning electron microscope (JEOL JSM-7401F).

### Annotation of the *Vibrio* sp. dhg genome sequence

To construct a draft of the *Vibrio* sp. dhg genome, purified genomic DNA (gDNA) was treated with the SMRT cell 8Pac v3 and DNA Polymerase Binding Kit P6 (Pacific Biosciences, CA, USA) and analyzed using the PacBio sequencer at Macrogen (Seoul, Korea). We obtained a total of 147,285 reads for 1,302,603,996 bases and then de novo assembled the reads using RS HGAP Assembly (v3.0) with default options. To correct for errors in the draft genome generated by the PacBio sequencer, we additionally used the Illumina short-read sequencer. A gDNA library was prepared using the KAPA HyperPlus Kit according to the manufacturer’s instructions (KAPA Biosystems, Wilmington, MA, USA). This short gDNA library was sequenced using the MiniSeq 300-cycle mid-output kit (Illumina, San Diego, CA, USA). Paired-end reads obtained from MiniSeq were mapped onto the assembled genome from PacBio data using the breseq pipeline^35^ with default options. Predicted mutations were further validated by Sanger sequencing. All errors were listed in Table S5. The final versions of each contig were uploaded to GenBank (accession numbers: CP028943, CP028944, and CP028945). The re-sequenced genome was annotated using Rapid Annotations of Subsystem Technology^36^.

### RNA-Seq

For expression profiling, *Vibrio* sp. dhg was cultured in buffered minimal medium supplemented with alginate, mannitol, or glucose as a sole carbon source, and harvested at early exponential phase (OD_600_ = 0.6). Total RNA including small RNAs was extracted by treating the cells with RNAprotect Bacteria Reagent (Qiagen, Hilden, Germany) and using an RNeasy Plus Mini kit (Qiagen, Hilden, Germany) according to the manufacturer’s instructions. These samples were prepared in biological duplicates. The concentrations of total RNA were measured using a Thermo NanoDrop One spectrophotometer (Waltham, MA, USA) and the quality of the samples was assessed using a Bioanalyzer with the RNA 6000 Pico kit from Agilent Technologies (Santa Clara, CA, USA). Then, the ribosomal RNAs were removed from the total RNA using the Ribo-Zero rRNA Removal Kit for Gram-negative bacteria (Illumina, San Diego, USA). The quality of the rRNA-free samples was also checked using the Bioanalyzer with the RNA 6000 Pico kit. The strand-specific RNA-seq library was constructed using the KAPA Stranded RNA-Seq Library Preparation Kit for Illumina platforms according to the manufacturer’s instructions. The size distribution of the complementary DNA library was assessed using the Bioanalyzer with a High Sensitivity DNA kit from Agilent Technologies. The samples were sequenced using the MiniSeq 75-cycle High-output Kit (Illumina, San Diego, USA) according to the manufacturer’s instructions. The quality of the sequence reads obtained from RNA-seq was checked by FastQC^37^, and then aligned to the modified reference genome using bowtie2^38^. The Cufflink package (http://cufflinks.cbcb.umd.edu/)^39^ was run to obtain transcriptomic data.

### Consensus promoter sequence annotation

To identify consensus promoter sequences of endogenous genes, we piled up the mRNA fragments from RNA-Seq reads. The mapped reads were extended to the fragment length and accumulated. For mapping, we used bowtie2 with default parameters^38^. Regions containing transcription start sites were extracted from the pile-up distribution for each gene. We manually inspected the upstream sequences of ribosomal genes and identified the promoter sequences. All the inspected promoter sequences were then aligned by CLUSTALW and a sequence logo was generated using Web logo (https://weblogo.berkeley.edu/logo.cgi).

### Genetic engineering of *Vibrio* sp. dhg

Purified plasmids were transformed into *Vibrio* sp. dhg strain via a previously established electroporation method^10^. Briefly, a single colony was inoculated into 3 mL of LBv2 medium. When the medium became turbid, the culture was then transferred into 10 mL of fresh medium using a 1/100 dilution. When an OD_600_ of 0.6 was reached, the culture was incubated in an icebox for 10 min. Subsequently, a cell pellet was prepared by centrifugation at 13,000 × *g* for 1 min at 4 °C. This pellet was washed twice with electroporation buffer and resuspended to achieve an OD_600_ of 16. Then, more than 100 ng of plasmid DNA was added to 90 μL of cell suspension and 0.8 kV was applied for 2.5 ms using a micropulse-electroporator (Bio-Rad Laboratories, Richmond, CA, USA). The cells were recovered by addition of 1 mL of BHI recovery medium and incubated at 37 °C. Finally, the cells were spread on antibiotic-containing LBv2 agar plates. Plasmid introduction was confirmed by agarose gel or colony PCR using primers that specifically anneal to the plasmid DNA.

For genome engineering, we first made electro-competent cells using the cells expressing SXT recombinases (following the same protocol used for plasmid transformation). During sub-culture, 1 mM of IPTG was included. For allelic exchange, more than 10 μg of the dsDNA fragment was added to the cell suspension. The cells were recovered by incubating in BHI recovery medium for 3 h at 37 °C. Then, cells were spread on an agar plate containing 10 mg L^−1^ of chloramphenicol. The integrated selection marker was removed via the activity of FLP flippase^19^. The change of target locus was confirmed by colony PCR and Sanger sequencing.

### Fluorescence measurements

To measure the fluorescence of each strain, colonies were inoculated into 180 μL of buffered minimal medium containing 4 g L^−1^ of glucose using BioscreenC (Growth Curves, Helsinki, Finland). After two rounds of subculture, the fluorescences of 100-μL samples were analyzed using the VICTOR^3^ 1420 Multilabel Plate Reader (Perkin Elmer, MA, Waltham, USA). Specific fluorescence was calculated by dividing the fluorescence by the OD_600_. For inducible promoters, different concentrations of inducers were included at time zero (1 of mM IPTG, 100 μg L^−1^ of aTc, or 10 g L^−1^ of arabinose).

### Synthetic promoter strength prediction

To construct a predictive model from the synthetic promoters, we first converted the promoter sequences into a set of binary vectors. Each nucleotide was set as (1, 0, 0, 0), (0, 1, 0, 0), (0, 0, 1, 0), or (0, 0, 0, 1) for ‘A’, ‘C’, ‘G’, or ‘T’, respectively. Then, we used the Ridge regression model to determine the coefficients and predict promoter strengths. The Ridge regression model addresses the ordinary least squares problem to determine the coefficients that minimize a residual sum of squares and the size of coefficients as shown below:

1 $$\min \left( {||Xw - y||_2^2} + \alpha ||w||_2^2\right)$$

where *X* denotes the vector-transformed promoter sequences, *y* denotes the promoter strengths, and *w* is a coefficient vector. *α* is a complexity parameter that balances the weight of emphasis given to minimizing the residual sum of squares and minimizing the sum of the square of coefficients. We performed leave-one-out cross-validation (LOOCV) in order to estimate how accurately the predictive model performs in practice. For each synthetic promoter sequence, we made a predictive model with the other sequences as a training set (i.e., the target sequence was excluded). For the implementation, we used the linear_model module from the sklearn package of python.

### Biochemical production

Biochemical production (ethanol, 2,3-BDO, and lycopene) from mimetic sugar mixture was conducted using the buffered minimal medium, however, the concentration of NaCl was reduced to 10 g L^−1^. In the case of 2,3-BDO, an additional 5 g L^−1^ of yeast extract was included in the medium. The medium was supplemented with 30 g L^−1^ of an alginate-mannitol mixture (1:2 ratio) as carbon source. When 80 g L^−1^ of sugar was provided, 30 g L^−1^ and 20 g L^−1^ of the sugar mixture were additionally added at 6 h and 12 h. Cells were cultivated in either 50 mL (for ethanol production) or 25 mL (for 2,3-BDO and lycopene production) of medium in a 350-mL flask at 200 rpm.

For ethanol production from brown macroalgae, a powder of kelp (60 mesh) harvested in Korea was purchased from a local market. Its sugar composition was quantified using 10 g L^−1^ of the kelp solution with water. Culture was conducted in the bioreactor containing the 1 L buffered medium with 10 g L^−1^ of alginate and mannitol (1:2) as initial carbon sources. Especially, phosphate buffer concentration was adjusted to 20 mM and 5 g L^−1^ of yeast extract were additionally provided. Sterile air at 2 L min^−1^ was provided during initial 6 h. Then, the cells were cultured anaerobically and 20 g L^−1^ of the kelp powder was added with a 3 h interval. The culture was continuously stirred at 300 rpm.

### Metabolite quantification

Sugars and metabolites (including ethanol and 2,3-BDO) were quantified using the UltiMate^TM^ 3000 analytical high-performance liquid chromatography system (Dionex, Sunnyvale, CA, USA) equipped with an Aminex HPX-87H column (Bio-Rad Laboratories). As a mobile phase, we used 5 mM of sulfuric acid at a flow rate of 0.6 mL min^−1^ and 65 °C. The refractive index (RI) signal was monitored using a Shodex RI-101 detector (Shodex, Klokkerfaldet, Denmark).

Alginate was quantified using a modified version of a previously described method^40^. Briefly, 200 μL of the sample was mixed with 1 mL of 0.025 M sodium tetraborate·10 H_2_O in sulfuric acid. After cooling the mixture in an icebox, 40 μL of 0.125% carbazole in absolute ethanol was added and the mixture gently was shaken. The absorbance at 530 nm was measured using the Hidex Sense microplate reader. The samples were appropriately diluted to fall within a 0–2 g L^−1^ range and a standard curve was prepared with using various concentrations of alginate solution (0, 0.05, 0.2, 0.5, 1, and 2 g L^−1^).

To quantify lycopene production, lycopene from cell pellets was extracted by adding pure acetone. The absorbance of the samples was then measured at 475 nm^41^.

### Pathogenicity analysis

Potential pathogenicity of *Vibrio*. sp dhg was evaluated by searching known *Vibrio* toxins (Ace, CT, MARTX, TDH, TRH, VCC, and Zot) using the Basic Local Alignment Search Tool (BLAST, https://blast.ncbi.nlm.nih.gov/Blast.cgi?PROGRAM=tblastn)^10,42,43^. Amino acid sequence of the toxins and genome sequence of *Vibrio* sp. dhg were used as inputs.

### Reporting summary

Further information on research design is available in the Nature Research Reporting Summary linked to this article.

## Supplementary information

Supplementary Information

Peer Review File

Reporting Summary

Description of Additional Supplementary Files

Supplementary Data 1

Supplementary Data 2

Supplementary Data 3

Supplementary Data 4

Source Data

## Data Availability

Data supporting the findings of this work are available within the paper and its Supplementary Information files. A reporting summary for this Article is available as a Supplementary Information file. The genome sequence of *Vibrio* sp. dhg has been uploaded to Genbank under accession number CP028943, CP028944, and CP028945. The transcriptomic data was deposited in Gene Expression Omnibus under accession number GSE119357. A reporting summary for this article is available as a Supplementary Information file. The source data underlying Figs. 1b–d, 2a–c are provided as a Source Data file. The other datasets generated and analyzed during the current study are available from the corresponding authors upon request.
