# Supplementary material for: Vibrio sp. dhg as a platform for the biorefinery of brown macroalgae
Source: Nat Commun. 2019 Jun 6;10:2486. doi: 10.1038/s41467-019-10371-1 (PMC6554313; doi:10.1038/s41467-019-10371-1)
Supplement: Supplementary file 1 — Supplementary Information [file 41467_2019_10371_MOESM1_ESM.pdf]

Vibrio sp. dhg as a platform for the biorefinery of brown macroalgae

Lim *et al.*

Supplementary Note 1. Buffer and medium composition

Alginate minimal medium[1]: 4 g L^{-1} alginic acid sodium salt from brown algae (Sigma, A2033, CAS number 9005-38-3), 5 g L^{-1} $(NH_4)_2SO_4$, 30 g L^{-1} NaCl, 2 g L^{-1} K_2HPO_4, 0.5 g L^{-1} $MgSO_4 \cdot 7H_2O$, and 2 mL L^{-1} trace metal solution.

Buffered minimal medium: 4 g L^{-1} carbon source, 5 g L^{-1} $(NH_4)_2SO_4$, 30 g L^{-1} NaCl, 10.7 g L^{-1} K_2HPO_4, 5.2 g L^{-1} KH_2PO_4, 0.5 g L^{-1} $MgSO_4 \cdot 7H_2O$, and 2 mL L^{-1} trace metal solution.

M9 medium: 4 g L^{-1} glucose, 12.8 g L^{-1} $Na_2HPO_4 \cdot 7H_2O$, 3 g L^{-1} KH_2PO_4, 1 g L^{-1} NH_4Cl, 0.5 g L^{-1} NaCl, 0.5 g L^{-1}, 0.5 g L^{-1} $MgSO_4 \cdot 7H_2O$, 1 mM $CaCl_2$.

CG medium (modified from CGXII medium): 4 g L^{-1} glucose, 5 g L^{-1} $(NH_4)_2SO_4$, 5 g L^{-1} Urea, 1 g L^{-1} KH_2PO_4, 1 g L^{-1} K_2HPO_4, 0.25 g L^{-1} $MgSO_4 \cdot 7H_2O$, 36 μM $FeSO_4$, 68 μM $CaCl_2$, 0.01 g L^{-1} $MnSO_4 \cdot H_2O$, 3.48 μM $ZnSO_4$, 0.2 mg L^{-1} $CuSO_4 \cdot 5H_2O$, 0.084 μM $NiCl_2$, 1.9 mM protocatechuic acid, 0.2 mg L^{-1} biotin, 2 mg L^{-1} thiamine hydrochloride

SC medium: 4 g L^{-1} glucose and 6.7 g L^{-1} Yeast Nitrogen Base without amino acids (Sigma Y0626).

LB medium[2]: 10 g L^{-1} tryptone, 5 g L^{-1} yeast extract, and 10 g L^{-1} NaCl.

LBv2 medium[2]: 10 g L^{-1} tryptone, 5 g L^{-1} yeast extract, 10 g L^{-1} NaCl, and v2 salts.

BHI medium: 37 g L^{-1} Brain Heart Infusion (BHI, BD).

BHIv2 medium[2]: 37 g L^{-1} BHI and v2 salts.

YPD medium: 20 g L^{-1} peptone and 10 g L^{-1} yeast extract.

BHI recovery medium: 680 mM sucrose, 37 g L^{-1} Brain Heart Infusion powder, and v2 salts.

v2 salts[2]: 11.92 g L^{-1} NaCl, 0.3 g L^{-1} KCl, and 2.2 g L^{-1} $MgCl_2$.

Trace metal solution (ATCC MD-TMS): 0.5 g L^{-1} ethylenediaminetetraacetic acid, 3.0 g L^{-1} $MgSO_4 \cdot 7H_2O$, 0.5 g L^{-1} $MnSO_4 \cdot H_2O$, 1.0 g L^{-1} NaCl, 0.1 g L^{-1} $FeSO_4 \cdot 7H_2O$, 0.1 g L^{-1} $Co(NO_3)_2 \cdot 6H_2O$, 0.1 g L^{-1} $CaCl_2$ (anhydrous), 0.1 g L^{-1} $ZnSO_4 \cdot 7H_2O$, 0.01 g L^{-1} $CuSO_4 \cdot 5H_2O$, 0.01 g L^{-1} $AlK(SO_4)_2$ (anhydrous), 0.01 g L^{-1} H_3BO_3, 0.01 g L^{-1} $Na_2MoO_4 \cdot 2H_2O$, 0.001 g L^{-1} Na_2SeO_3 (anhydrous), 0.01 g L^{-1} $Na_2WO_4 \cdot 2H_2O$, and 0.02 g L^{-1} $NiCl_2 \cdot 6H_2O$.

Electroporation buffer: 680 mM sucrose and 7 mM K_2HPO_4 (pH was adjusted by adding 5 M HCl).

pH of all media and a buffer was adjusted to 7. Agar plates were prepared by including 15 g L^{-1} of agar into media.

Supplementary Note 2. Simultaneous assimilation of alginate and mannitol for efficient ethanol production

$$\text{DEHU} + 0\ \text{NAD}^+ + 1\ \text{ADP} + 1\ \text{P}_i \rightarrow 2\ \text{Pyruvate} + 0\ \text{NADH} + 0\ \text{H}^+ + 1\ \text{ATP} \quad (\mathbf{1})$$

$$\text{Mannitol} + 3\ \text{NAD}^+ + 2\ \text{ADP} + 2\ \text{P}_i \rightarrow 2\ \text{Pyruvate} + 3\ \text{NADH} + 3\ \text{H}^+ + 2\ \text{ATP} \quad (\mathbf{2})$$

$$2\ \text{Pyruvate} + 2\ \text{NADH} + 4\ \text{H}^+ \rightarrow 2\ \text{Ethanol} + 2\ \text{NAD}^+ + 2\ \text{CO}_2 \quad (\mathbf{3})$$

If only DEHU is used, production of ethanol should be low due to the lack of reducing equivalent (NADH). Sacrifice of carbon in the citric acid cycle is needed to provide additional reducing equivalent. In case of mannitol assimilation, excess reducing equivalent is generated, hindering regeneration of NAD in oxygen-limited conditions. This redox imbalance also significantly reduces ethanol production by poor mannitol catabolism[3].

$$\text{DEHU} + 2\ \text{Mannitol} + 6\ \text{NAD}^+ + 5\ \text{ADP} + 5\ \text{P}_i \rightarrow 6\ \text{Pyruvate} + 6\ \text{NADH} + 6\ \text{H}^+ + 5\ \text{ATP} \quad (\mathbf{4})$$

$$6\ \text{Pyruvate} + 6\ \text{NADH} + 12\ \text{H}^+ \rightarrow 6\ \text{Ethanol} + 6\ \text{NAD}^+ + 6\ \text{CO}_2 \quad (\mathbf{5})$$

When alginate and mannitol are used simultaneously in a 1:2 ratio, stoichiometric balance of the reducing cofactor is achieved. This enables efficient ethanol production without additional carbon loss.

Supplementary Note 3. Plasmid cloning procedures

Plasmids were prepared by conventional restriction enzyme methods, blunt-end ligation, or the Gibson assembly method[4] using the NEBuilder® HiFi DNA assembly cloning kit (NEB, Ipswich, USA). Coding sequences of *sgfp* and *tetR* were synthesized using the gBlock® gene fragment synthesis service (Integrated DNA Technologies, Skokie, USA). Mach -T1® was used as a cloning host.

To construct the pACYC_VP15_sgfp plasmid, the *sgfp* gene was amplified using the sgfp_VP15_F1, sgfp_VP15_F2, sgfp_VP15_F3, and sgfp_B primers with the synthesized *sgfp* coding sequence as a template. The amplified fragment was inserted into the *Pst*I-*EcoR*I site of the pACYC_duet plasmid.

To prepare the pACYC_Plac_sgfp plasmid, the pACYC_VP15_sgfp plasmid was amplified using the sgfp_Plac_Ptac_F and sgfp_Plac_B primers. Then, the amplified fragment was self-circularized by blunt-end ligation. In the case of pACYC_Ptac_sgfp, the sgfp_Ptac_B primer was used instead of the sgfp_Plac_B primer.

For the construction of the pACYC_PT7_sgfp plasmid, the T7 RNAP gene was amplified from the genomic DNA of BL21(DE3) using the T7RNAP_Plac_F1, T7RNAP_Plac_F2, T7RNAP_Plac_B1, and T7RNAP_Plac_B2 primers. The vector fragment was amplified using the pACYC_T7_F and pACYC_T7_B primers with pACYC_duet as a template. These two fragments were assembled using Gibson assembly to yield the pACYC_PT7 plasmid. Subsequently, the *sgfp* gene was amplified with the sgfp_PT7_F1 and sgfp_PT7_F2, sgfp_PT7_B primers and the pACYC_VP15_sgfp plasmid inserted into the *EcoR*I-*Xho*I site of the pACYC_PT7 plasmid. It should be noted that the native *lacI* expression cassette from *E. coli* was used.

To construct the pACYC_Ptet_sgfp plasmid, pACYC_VP15_sgfp was amplified using the sgfp_Ptet_F and sgfp_Ptet_B primers and self-circularized by blunt-end ligation. Then, the *tetR* gene amplified with the tetR_VP13_F and tetR_VP13_B primers and the purchased *tetR* fragment were inserted into the *Eco*RI-*Nco*I site of the resulted plasmid. This *tetR* expression cassette contains a VP13 (P_{J23100}) promoter and a mid-low strength 5′-UTR (Supplementary Table 8).

The pACYC_Para_sgfp plasmid was constructed by combining the *araC*-P_{ara} fragment, amplified using the araC_Para_F and araC_Para_B primers with the pKD46 plasmid as a template, and the vector fragment, amplified using the pACYC_Para_vec_F and pACYC_Para_vec_R primers with pACYC_VP15_sgfp as a template. The used *araC*-P_{ara} fragment in the pKD46 plasmid was originated from *E. coli* strain.

Synthetic promoter library plasmids were prepared by self-circularization of an amplified PCR product using sgfp_prom_lib_F and sgfp_prom_lib_B primers with pACYC_VP15_sgfp as a template. 5′-UTR library plasmids were constructed using sgfp_utr_lib_F and sgfp_utr_lib_B primers.

The pACYA plasmid was assembled by combining a vector fragment amplified from pACYC_Duet using the pACYC_vec_F and pACYC_vec_B primers, with a *bla* gene fragment amplified from the pUC19 plasmid using the primers Amp_F and Amp_B.

For the pACYA_SXT plasmid, the genes for Red recombination were initially cloned into the pACYCA plasmid to yield the pACYCA_Red plasmid. More specifically, the vector fragment was amplified from the pACYCA plasmid using primers pACYA_vec_F and B and the Red gene fragment was amplified using the primers Red_F and B with the pKD46 as a template. These two fragments were assembled together. Subsequently, the original *exo* and *beta* genes in the pACYA_red plasmid were replaced by the genes encoding SXT

recombinase to yield the pACYA_SXT plasmid. For this modification, two fragments – a vector fragment amplified from the pACYCA_red plasmid using primers pACYC_SXT_F and B and an SXT gene fragment amplified from purified genomic DNA of VDHG using the primers SXT_F and B were assembled together.

To construct the pRSF_FLP plasmid for expression of FLP flippase from *S. cerevisiae*[5], the *flp* gene was amplified using FLP_F1, FLP_F2, and FLP_B primers with pCP20 as a template. Next, the amplified fragment was inserted into the *Sac*I and *Not*I sites in the pRSFduet plasmid.

The plasmids for gene deletion (pCDF-dns, pCDF-ldhA, pCDF-frd, pCDF-pflB, and pCDF-orf) were prepared by Gibson assembly in which the *cat* gene fragment was flanked by the homology arms of each corresponding target sequence. Vector fragments were amplified by using the Gibson_[gene name]_1F and Gibson_[gene name]_1B primers with the pCDF_duet plasmid as the template. Upper and lower homology sequences were amplified from purified *Vibrio* sp. dhg genomic DNA using the Gibson_[gene name]_2F, Gibson_[gene name]_2B, Gibson_[gene name]_4F, and Gibson_[gene name]_4B primers. The *cat* gene was amplified by using the primers Gibson_[gene name]_3F and Gibson_[gene name]_3B with FRT72_cat or FRTwt_cat as the template.

For ethanol production, the pACYC_EtOH plasmid was constructed by assembling together *pdc*, *aldB*, and vector fragments. The *pdc* and *aldB* fragments were amplified using the Pdc_F1, Pdc_F2, Pdc_B, AldB_F1, AldB_F2, and AldB_B primers and gDNA from *Zymomonas mobilis*. The vector fragment was amplified using the pACYC_EtOH_F and pACYC_EtOH_B primers with the pACYC_Duet plasmid as a template.

The pACYC_BDO plasmid was constructed to overexpress the *budABC* operon from *Enterobacter aerogenes* for 2,3-BDO production. Initially, the *budABC* operon was amplified

from the pZSbudABC plasmid using the primers Bud_F1, Bud_F2, and Bud_B. Then, this fragment was assembled with a vector fragment amplified from pACYC_Duet using the pACYC_BDO_F and pACYC_BDO_B primers.

Lycopene plasmids (pACYC_Lyc, pACYC_Lyc2, and pACYC_Lyc3) were prepared by restriction enzyme digestion. Specifically, to obtain the pACYC_Lyc plasmid, the *crtEBI* cassette of the pCDF_idi_ispA_crtEBI plasmid was inserted into *Nco*I-*Hind*III cloning site of pACYC_duet. For the construction of the pACYC_lyc2 plasmid, expression cassettes of the *idi*, *ispA*, and *crtEBI* genes were inserted into the *Nco*I-*Mfe*I cloning site of pACYC_duet. The expression cassette for *dxs* (from pACYC_dxs) was inserted into the *Kpn*I-*Pac*I cloning site of the pACYC_lyc2 plasmid to obtain the pACYC_lyc3 plasmid.

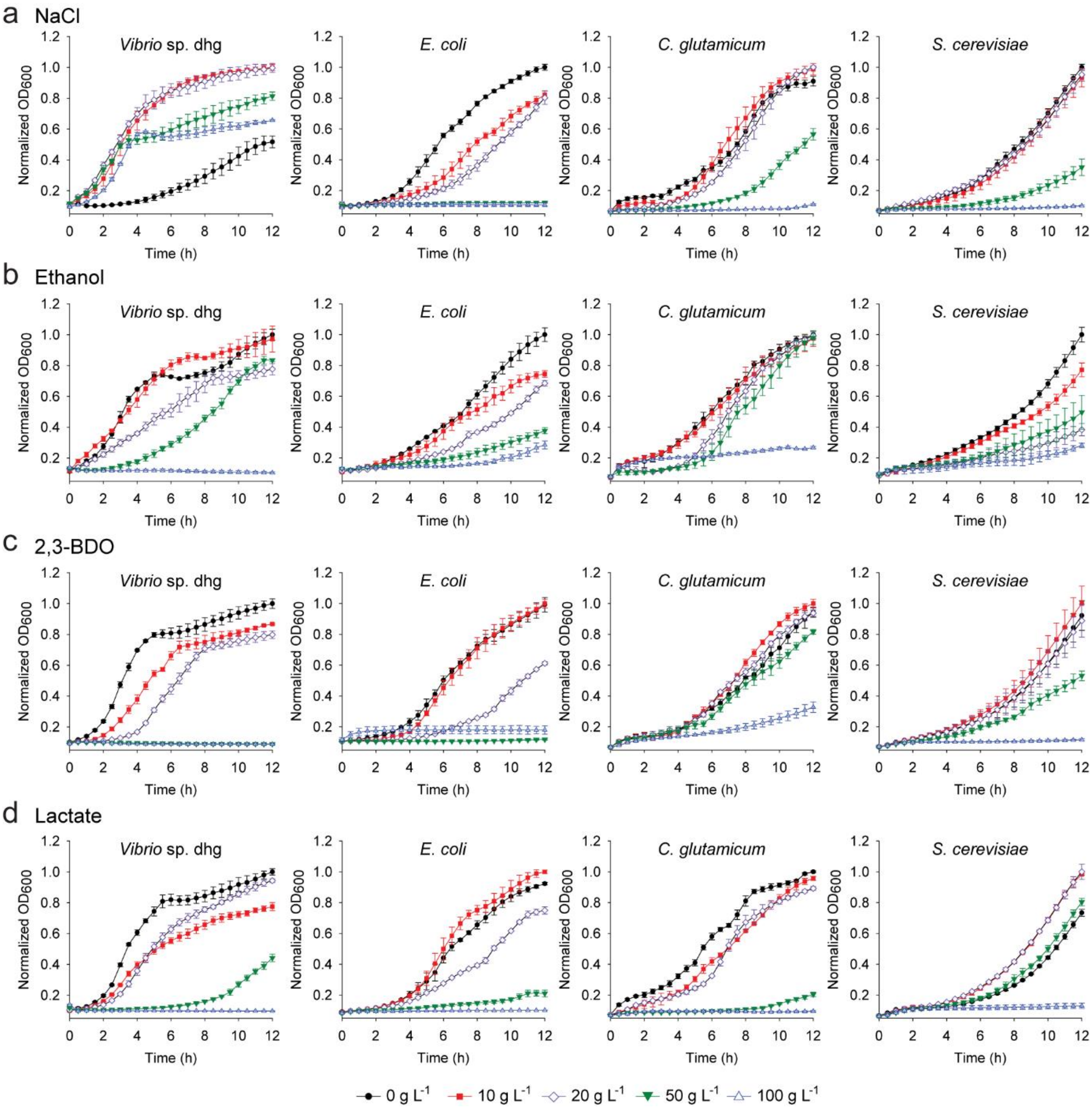

Supplementary Figure 1. Tolerance comparison of *Vibrio* sp. dhg with other microbial hosts

Growth curves of *Vibrio* sp. dhg, *E. coli*, *C. glutamicum*, and *S. cerevisiae* under presence of different concentrations of **a**, NaCl, **b**, ethanol, **c**, 2,3-BDO, **d**, lactate (neutralized to pH 7) in the medium. Cells were grown in 100 μL medium contained in microtiter plate wells at 30 ^{o}C (37 ^{o}C for *E. coli*). OD_{600} was periodically measured by the plate reader and normalized by the maximum value of each experiment. Symbols: closed black circle, 0 g L^{-1}; closed red

square, 10 g L^{-1}; open purple diamond, 20 g L^{-1}; closed green downward triangle, 50 g L^{-1}; open blue upward triangle, 100 g L^{-1}. Error bar indicates the standard deviations of three independent cultures (*n*=3).

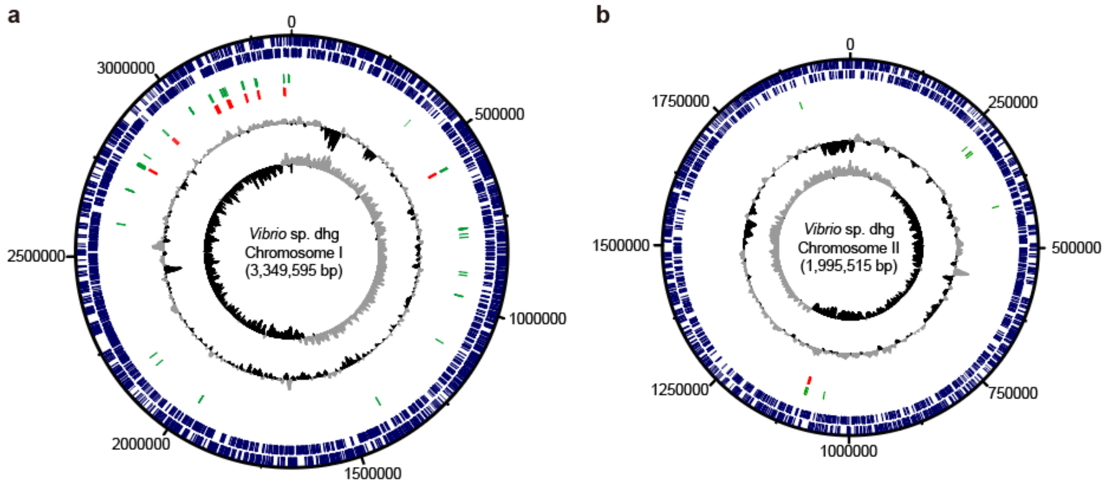

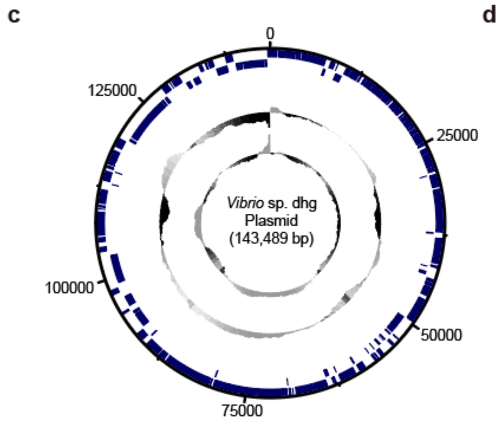

d

Name	Length	GC%	CDS	tRNA	rRNA	GenBank Accesion Number
Chromosome 1	3,349,595	45.2	2,982	117	34	CP028943
Chromosome 2	1,995,515	44.3	1,814	13	3	CP028944
Plasmid	143,489	42.0	174	0	0	CP028945
Total	5,488,599	44.9	4,970	130		

Supplementary Figure 2. Genome information of *Vibrio* sp. dhg

a-c, Circular maps of **a,** chromosome 1, **b**, chromosome 2, and **c**, plasmid. Starting from the outer circle, the forward coding sequence (dark blue), reverse coding sequence (dark blue), tRNA (green), rRNA (red), GC plot (black: below average, grey: above average), and GC skew (black: below average, grey: above average) are depicted. The images were drawn using DNAplotter (https://www.sanger.ac.uk/science/tools/dnaplotter)[6]. **d**, Annotated genome information, analysed by Rapid Annotations using the Subsystems Technology (RAST) server[7].

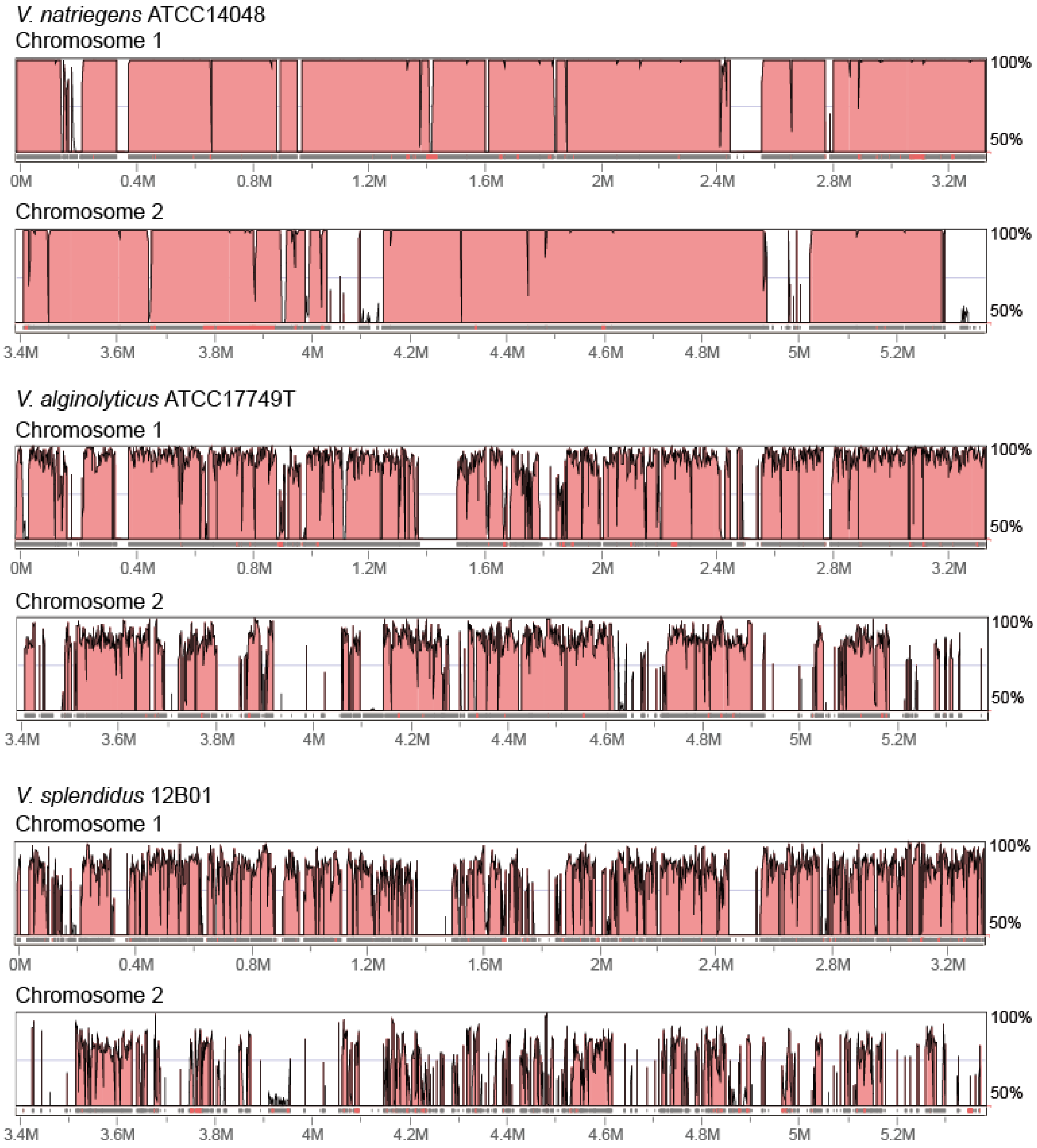

Supplementary Figure 3. Comparative genome analysis with other *Vibrio* strains

The chromosomal sequence of *Vibrio* sp. dhg was compared with the genome of *V. natriegens* (accession number: CP016345 and CP016346), *V. alginolyticus* ATCC17749T (accession number: CP006718 and CP006719)[8] and *V. splendidus* 12B01 (accession number: NZ_AAMR00000000) using wgVISTA (http://genome.lbl.gov/cgi-bin/WGVistaInput)[9,10] with default parameters. For *V. natriegens*, the average similarity of the aligned sequence was

98%. As the full genome sequence of *V. splendidus* 12B01 was unavailable, its partial genome sequence was used for comparison. The graph indicates conserved regions with their level of similarity.

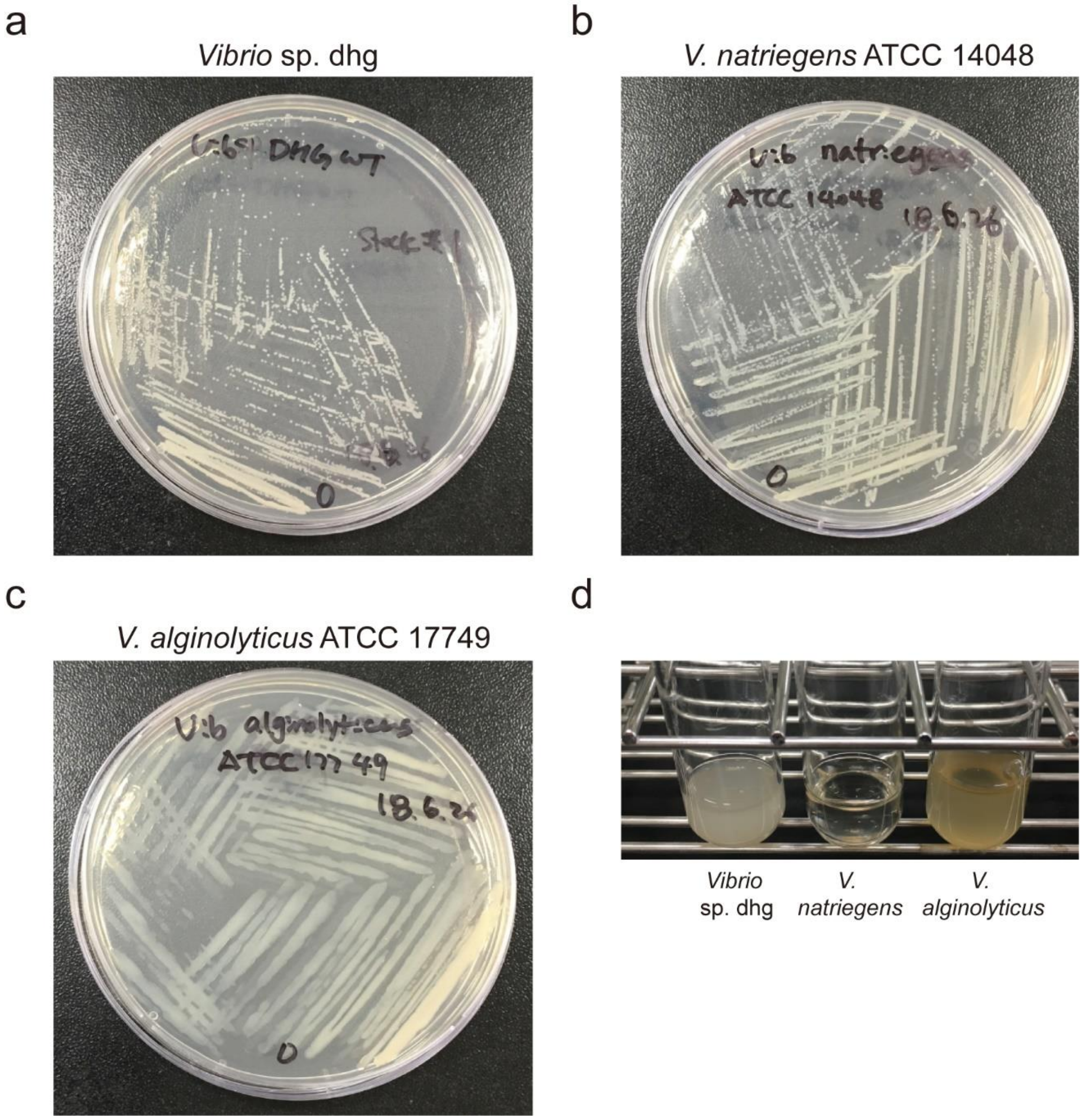

Supplementary Figure 4. Phenotypic comparison between *Vibrio* species

Colony formation on agar plates of **a,** *Vibrio* sp. dhg, **b,** *V. natriegens* ATCC14048 and **c,** *V. alginolyticus* ATCC17749. While both *Vibrio* sp. dhg and *V. natriegens* formed circular, convex colonies, *V. alginolyticus* formed irregular, flat colonies. **d,** Overnight cultures of the three strains in 3 mL of alginate minimal medium. *Vibrio* sp. dhg showed a superior ability to use alginate as the sole carbon source.

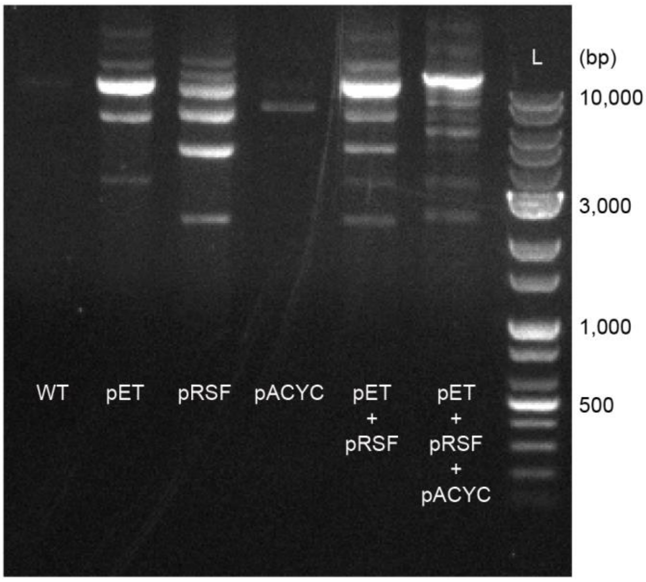

Supplementary Figure 5. Multiple plasmid maintenance in *Vibrio* sp. dhg

After extracting the plasmids (pET_duet, pRSF_duet, and pACYC_duet) from cells growing on antibiotic-containing 5-mL LB medium at 30 ^{o}C, 5 μL of the sample was run on a 1% agarose gel. Each plasmid showed different band intensities and different migration patterns. Antibiotic concentration: 100 μg mL^{-1} for ampicillin, 100 μg mL^{-1} for kanamycin and 10 μg mL^{-1} for chloramphenicol.

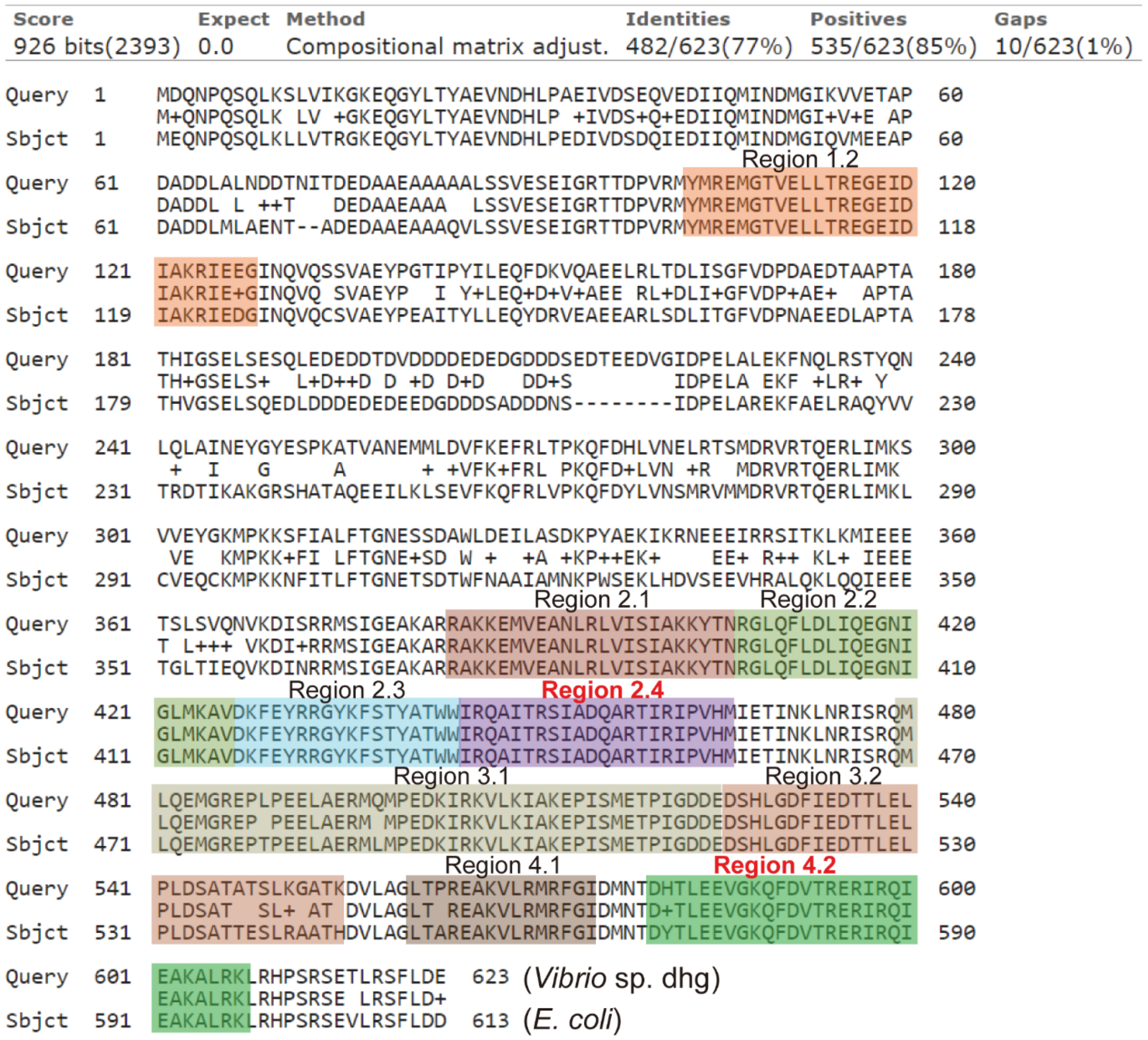

Supplementary Figure 6. Sequence alignment of sigma factor 70 from *Vibrio* sp. dhg and *E. coli*

Query sequence and subject sequence are the amino acid sequences of *Vibrio* sp. dhg σ_{70} (623 amino acids) and *E. coli* σ_{70} (613 amino acids), respectively. It is known that region 2.4 and 4.2 of *E. coli* σ_{70} contacts the -10 and -35 boxes of the promoter[11,12]. BLAST (https://blast.ncbi.nlm.nih.gov/Blast.cgi) was used for the sequence alignment (max score, 926; total score, 926; query cover, 100%; E-value, 0.0; percentage identity, 77.37%).

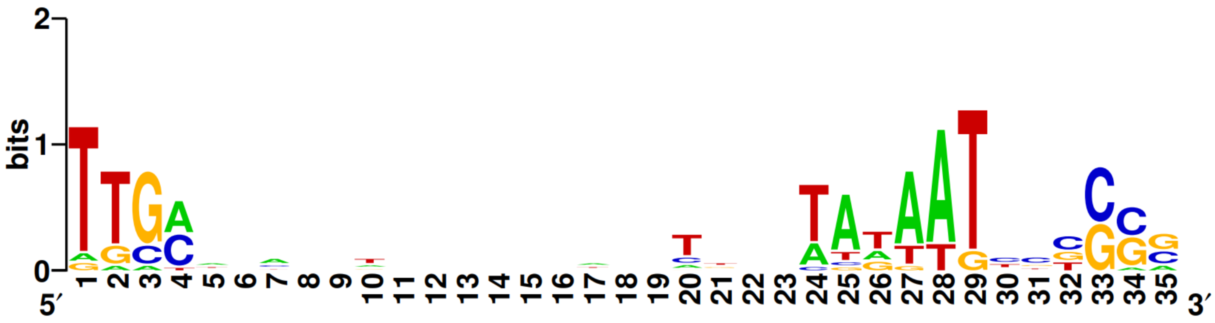

Supplementary Figure 7. Consensus promoter sequence of the ribosomal genes in *Vibrio* sp. dhg

A total of 15 promoter sequences were used to generate the consensus promoter sequence.

This image was generated using Weblogo (https://weblogo.berkeley.edu/logo.cgi).

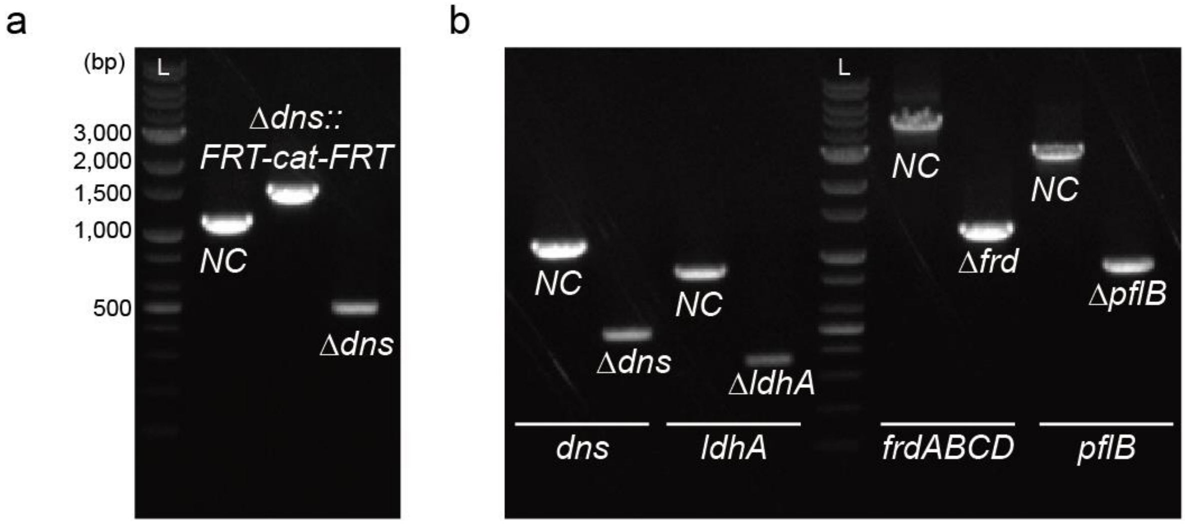

Supplementary Figure 8. Agarose gel electrophoresis to confirm genome editing

a, Deletion of the *dns* gene by insertion of the FRT-*cat*-FRT fragment. Lane 1: ladder; lane 2: negative control (1,118 bp); lane 3: after allelic exchange at the *dns* locus (1,512 bp); lane 4: after FLP flippase expression (494 bp). **b,** lane 1 and 2, deletion of *dns* (1,118 bp to 494 bp); lane 3 and 4, deletion of *ldhA* (870 bp to 362 bp); lane 5: ladder; lane 6 and 7, deletion of *frdABCD* (4,149 bp to 1,224 bp); and lane 8 and 9, deletion of *pflB* (2,801 bp to 839 bp). The same ladder was used for both gel electrophoresis experiments.

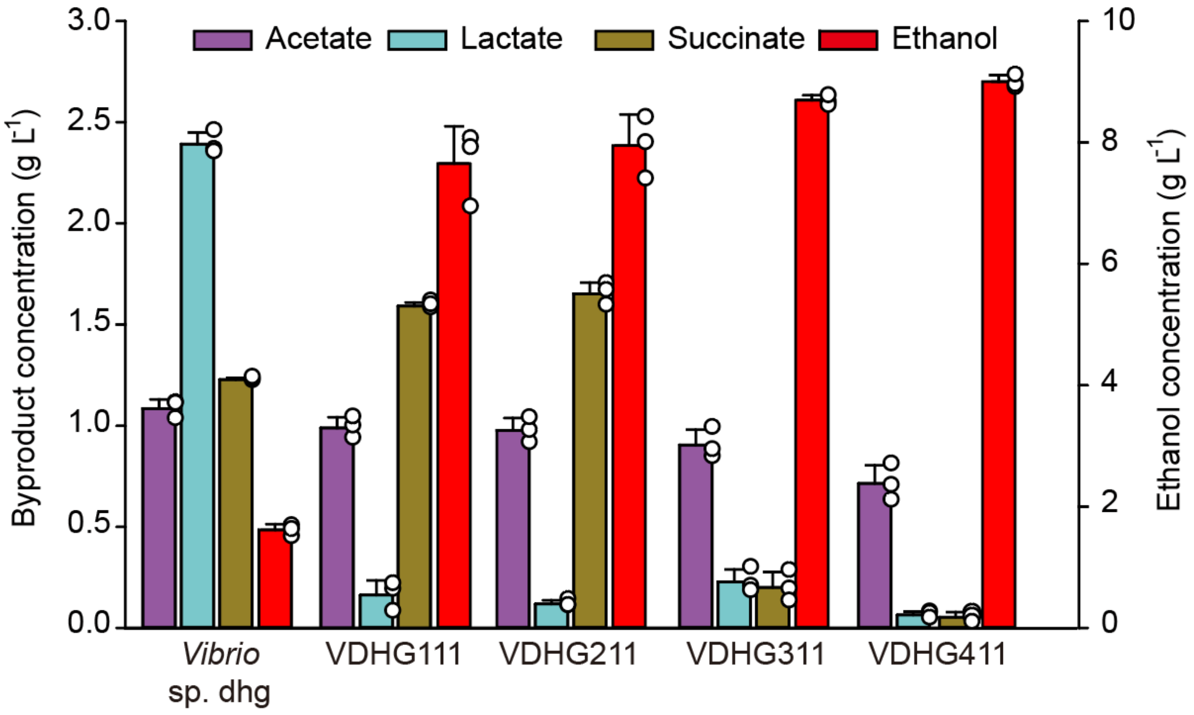

Supplementary Figure 9. Improved ethanol production in *Vibrio* sp. dhg via genome editing

The ethanol-producing *Vibrio* sp. dhg strains were cultivated in medium supplemented with 30 g L^{-1} of brown macroalgae sugar mixture (1:2 ratio of alginate to mannitol) during 12 h. Genotypes: *Vibrio* sp. dhg, the original isolated *Vibrio* sp. dhg; VDHG111, *Vibrio* sp. dhg Δ*dns* with pACYC_EtOH; VDHG211, *Vibrio* sp. dhg Δ*dns* Δ*ldhA* with pACYC_EtOH; VDHG311, *Vibrio* sp. dhg Δ*dns* Δ*ldhA* Δ*frdABCD* with pACYC_EtOH, VDHG411, *Vibrio* sp. dhg Δ*dns* Δ*ldhA* Δ*frdABCD* Δ*pflB* with pACYC_EtOH. Bars indicate metabolite concentrations (acetate, lactate, succinate, and ethanol). Error bar indicates the standard deviations of three independent cultures (*n*=3). Dot indicates actual data point.

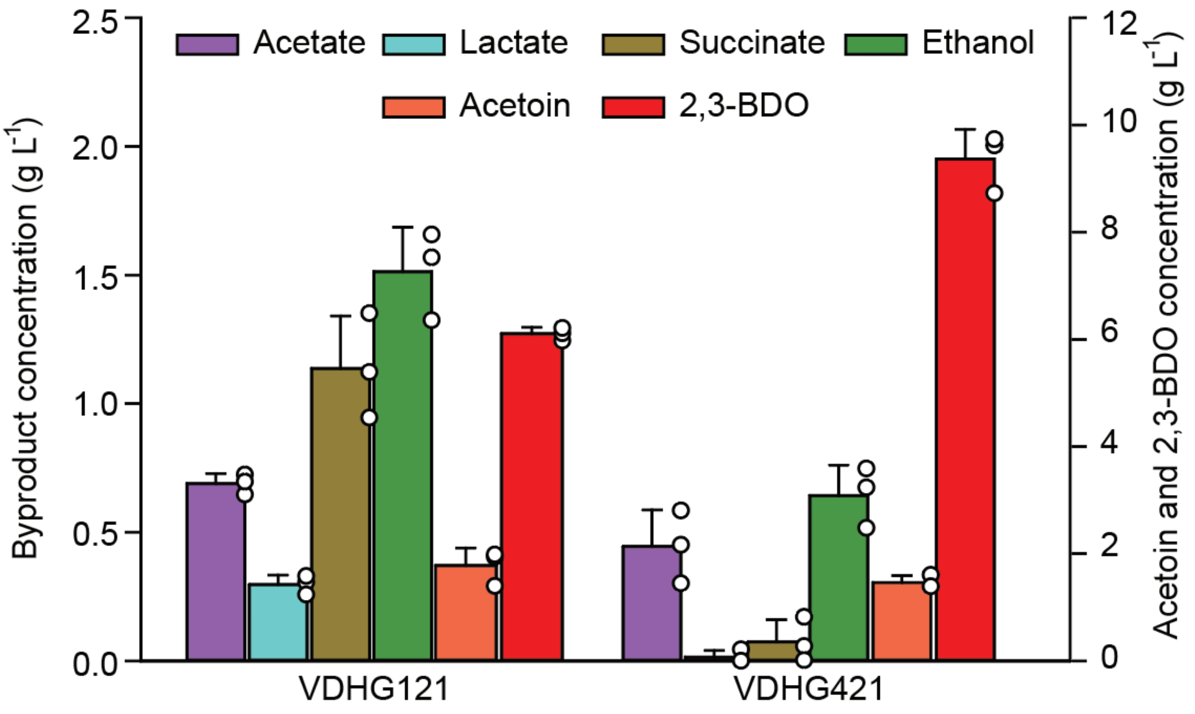

Supplementary Figure 10. Improved 2,3-BDO production in *Vibrio* sp. dhg via genome editing

The 2,3-BDO-producing *Vibrio* sp. dhg strains were cultivated in medium supplemented with 30 g L^{-1} of brown macroalgae sugar mixture (1:2 ratio of alginate to mannitol) during 9 h. Genotypes: VDHG121, *Vibrio* sp. dhg Δ*dns* with pACYC_BDO; VDHG421, *Vibrio* sp. dhg Δ*dns* Δ*ldhA* Δ*frdABCD* Δ*pflB* with pACYC_BDO. Bars indicate metabolite concentrations (acetate, lactate, succinate, ethanol, acetoin, and 2,3-BDO). Error bar indicates the standard deviations of three independent cultures (*n*=3). Dot indicates actual data point.

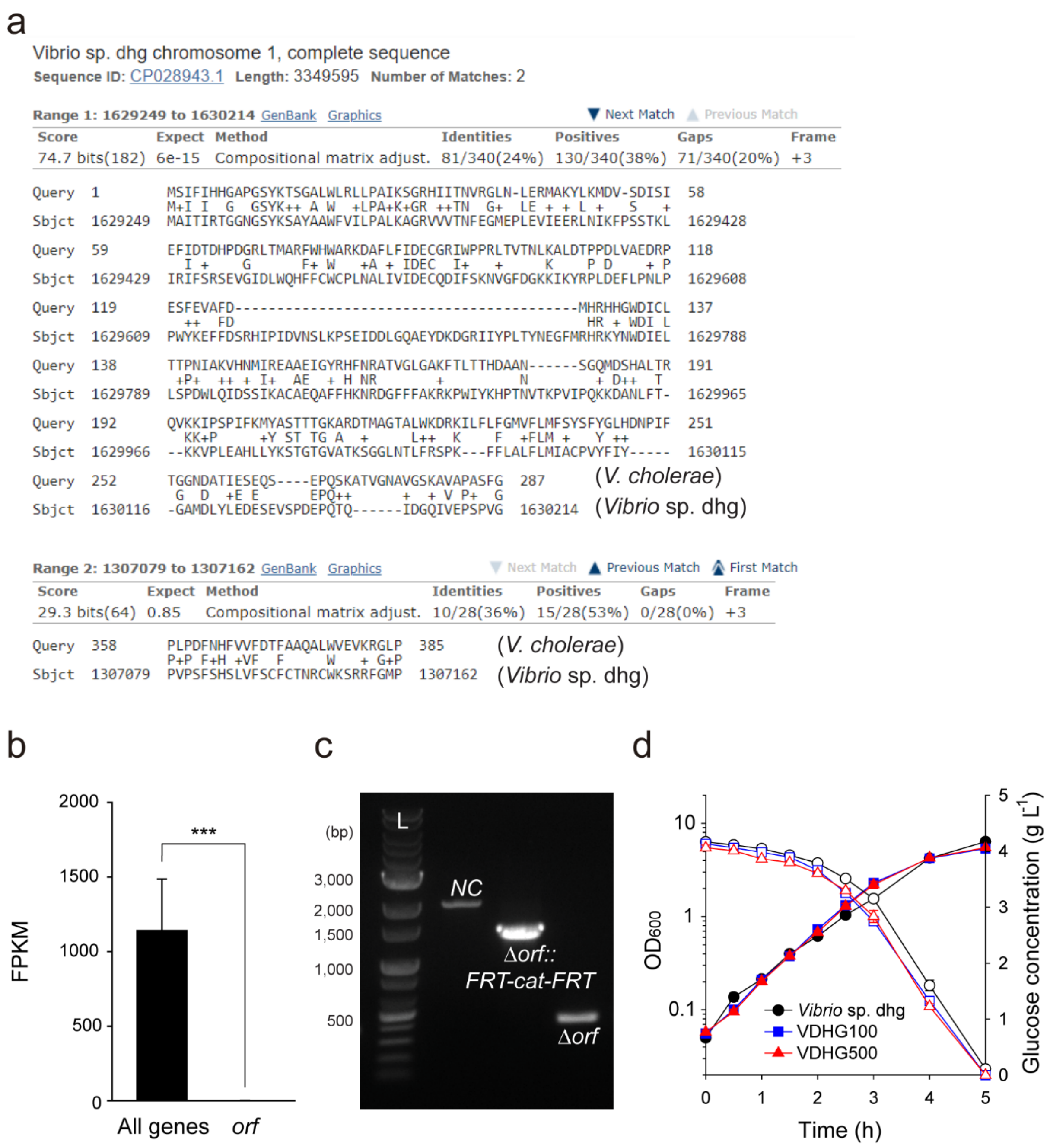

Supplementary Figure 11. Potential pathogenicity analysis of *Vibrio* sp. dhg

a, Sequence alignment of Zot protein (399 amino acids) from *V. chorlerae* and one open reading frame (ORF, 478 amino acids) from *Vibrio* sp. dhg. Query sequence and subject sequence are the amino acid sequences from *V. chorlerae* and *Vibrio* sp. dhg, respectively. BLAST (https://blast.ncbi.nlm.nih.gov/Blast.cgi) was used for the sequence alignment (max score, 74.7; total score, 103; query cover, 78%; E-value, 6e-15; percentage identity, 23.82%).

b, Comparison of fragments per kilobase per million (FPKM) values for all genes and the ORF gene. *** indicates $p < 0.001$ (two-sided *t*-test) **c,** agarose gel electrophoresis to confirm deletion of the ORF. Lane 1: Ladder; lane 2: negative control (1,977 bp); lane 3: after allelic exchange (1,412 bp); lane 4: after FLP flippase expression (394 bp). **d,** Comparison of the growth and the glucose consumption of *Vibrio* sp. dhg strain, the VDHG100 (Δ*dns*) strain (a parental strain for deletion), and the VDHG500 strain (Δ*dns* Δ*orf*). Closed symbols indicate OD_{600} and open symbols indicate glucose concentration for each strain. Black circle, *Vibrio* sp. dhg; blue square, the VDHG100 strain; red triangle, the VDHG500 strain. Error bar indicates the standard deviations of three independent cultures (n=3).

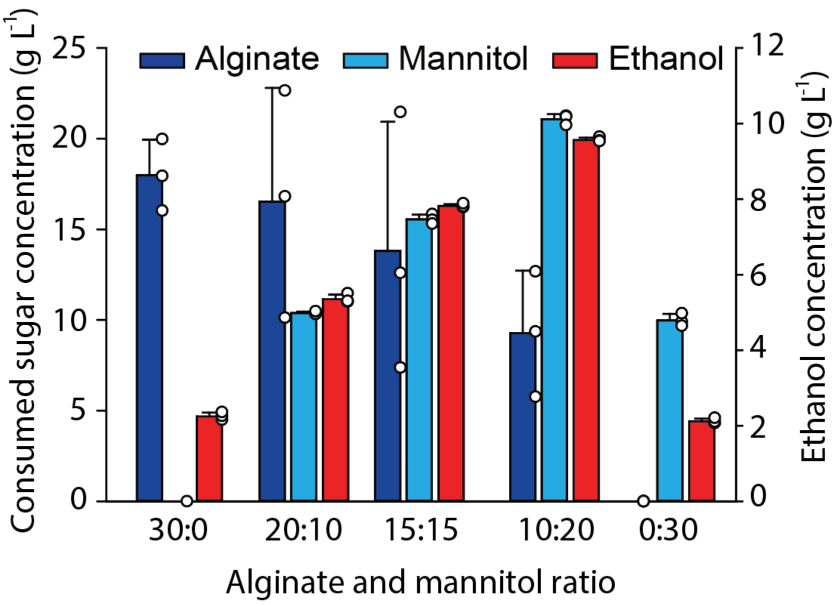

Supplementary Figure 12. Effect of sugar compositions to ethanol production

Amounts of consumed sugars and produced ethanol by the VDHG411 strain (*Vibrio* sp. dhg Δ*dns* Δ*ldhA* Δ*frdABCD* Δ*pflB* with pACYC_EtOH) with different sugar mixtures after 12 h. For the 30:0 experiment, only 20 g L^{-1} of alginate was supplied until 12 h due to 6-h interval in alginate feeding. Left two bars of each group indicate consumed alginate and mannitol concentration (g L^{-1}). Right bar indicates ethanol concentration (g L^{-1}). Error bar indicates the standard deviations of three independent cultures (*n*=3). Dot indicates actual data point.

Supplementary Table 1. Codon usage of *Vibrio* sp. dhg

Amino acid	Codon	RSCU[a]	Amino acid	Codon	RSCU[a]	Amino acid	Codon	RSCU[a]
Ala	GCU	0.900	Gly	GGU	1.000	Ser	UCU	1.000
Ala	GCC	0.543	Gly	GGC	0.707	Ser	UCC	0.335
Ala	GCA	1.000	Gly	GGA	0.273	Ser	UCA	0.827
Ala	GCG	0.926	Gly	GGG	0.217	Ser	UCG	0.522
Arg	CGU	1.000	Ile	AUU	1.000	Ser	AGU	0.784
Arg	CGC	0.630	Ile	AUC	0.847	Ser	AGC	0.811
Arg	CGA	0.355	Ile	AUA	0.222	Thr	ACU	0.967
Arg	CGG	0.087	Leu	UUA	0.898	Thr	ACC	1.000
Arg	AGA	0.246	Leu	UUG	0.832	Thr	ACA	0.832
Arg	AGG	0.078	Leu	CUU	0.825	Thr	ACG	0.841
Asn	AAU	0.800	Leu	CUC	0.409	Trp	UGG	1.000
Asn	AAC	1.000	Leu	CUA	0.597	Tyr	UAU	0.809
Asp	GAU	1.000	Leu	CUG	1.000	Tyr	UAC	1.000
Asp	GAC	0.644	Lys	AAA	1.000	Val	GUU	1.000
His	CAU	1.000	Lys	AAG	0.465	Val	GUC	0.549
His	CAC	0.974	Met	AUG	1.000	Val	GUA	0.654
Cys	UGU	1.000	Phe	UUU	1.000	Val	GUG	0.741
Cys	UGC	0.514	Phe	UUC	0.689	Stop	UAA	1.000
Gln	CAA	1.000	Pro	CCU	0.802	Stop	UAG	0.313
Gln	CAG	0.682	Pro	CCC	0.192	Stop	UGA	0.237
Glu	GAA	1.000	Pro	CCA	1.000			
Glu	GAG	0.537	Pro	CCG	0.561			

[a]The value was normalized by the maximum RSCU (Relative Synonymous Codon Usage)[13] value of the synonymous codons.

Supplementary Table 2. Modifications in the draft genome sequence of *Vibrio* sp. dhg

Position[a]	Modification	Position[a]	Modification
**Chromosome 1**		699,020	$(C)_{6\rightarrow7}$
13,057	+C	884,053	+G
209,742	$(G)_{7\rightarrow8}$	919,707	$(C)_{5\rightarrow6}$
573,005	$(G)_{5\rightarrow6}$	1,000,359	$(G)_{6\rightarrow7}$
799,862	+G	1,039,159	$(C)_{5\rightarrow6}$
1,015,310	C→G	1,090,859	A→T
1,076,817	+C	1,091,615	A→G
1,158,695	+G	1,092,279	C→A
1,162,133	$(C)_{5\rightarrow6}$	1,092,286	G→T
1,223,814	+C	1,092,641	T→A
1,343,347	+G	1,092,645	+A
1,382,633	+G	1,092,647	A→T
1,549,786	+G	1,093,739	A→G
1,746,429	A→G	1,094,597	$(C)_{5\rightarrow6}$
1,784,822	+G	1,271,725	+C
1,821,413	+GATT	1,345,040	+G
1,850,299	+G	1,471,064	$(G)_{5\rightarrow6}$
2,255,401	$(C)_{5\rightarrow6}$	1,714,482	$(G)_{5\rightarrow6}$
2,493,405	+[b]	1,730,271	$(G)_{7\rightarrow8}$
**Chromosome 2**		1,831,599	+C
22,916	G→C	**Plasmid**	
22,916	G→C	11,215	+G
339,259	+C	129,284	T→C
584,397	+G	129,560	G→A

[a]Position in the draft genome

[b]GAGCGATTGCGATCCGCAGACGAGTCAAAATAACTTCCGACTTGATGCTCGCTA

TTTTTTCCGCGACCACTTTTTGCTGTGGGGCAGAGTAACTGCGCGCGAGTGCTTC

CGCGCCAGACCAATCTTGCTGCTG

Supplementary Table 3. Physiological comparison with *Vibrio* sp. dhg and *V. natriegens*

Microorganism	Temperature (^oC)	Aeration	Culture type	Growth rate (h^{-1})	Biomass yield	Specific sugar uptake rate ($g\ g^{-1}$ DCW L^{-1})
Vibrio sp. dhg	30	Aerobic	Flask	1.16 ± 0.01	0.41 ± 0.02	2.84 ± 0.13
	30	Anaerobic	Serum bottle	0.66 ± 0.00	0.20 ± 0.02	3.36 ± 0.35
	37	Aerobic	Flask	0.99 ± 0.06	0.28 ± 0.02	3.51 ± 0.12
	37	Aerobic	Reactor[a]	1.55 ± 0.03	0.38 ± 0.10	4.38 ± 0.98
	37	Anaerobic	Serum bottle	1.04 ± 0.02	0.20 ± 0.01	5.19 ± 0.17
	37	Anaerobic	Reactor[a]	1.08 ± 0.27	0.22 ± 0.01	5.00 ± 1.46
V. natriegens	30	Aerobic	Flask	1.51 ± 0.01	0.45 ± 0.03	3.35 ± 0.24
	30	Anaerobic	Serum bottle	0.77 ± 0.04	0.21 ± 0.01	3.71 ± 0.09
	37	Aerobic	Flask	1.32 ± 0.15	0.41 ± 0.03	3.24 ± 0.24
	37	Aerobic	Reactor[a]	1.48 ± 0.06[b]	0.38 ± 0.03[b]	3.90 ± 0.08[b]
	37	Anaerobic	Serum bottle	1.20 ± 0.00	0.22 ± 0.00	5.36 ± 0.17
	37	Anaerobic	Reactor[a]	0.92 ± 0.01[b]	0.12 ± 0.01[b]	7.81 ± 0.71[b]

[a]For the reactor-scale experiments, 10 g L^{-1} of glucose was supplemented.

[b]Data from Hoffart *et al*[14].

Supplementary Table 4. Synthetic promoters for transcriptional control

Name	Sequence[a]	Relative strength[b]
Template	YTKAYRGCTAGCTCAGTCCTAGGKAYWRTGCTAGC	
VP1	CTTATGGCTAGCTCAGTCCTAGGGACAGTGCTAGC	0.024
VP2	TTTACGGCTAGCTCAGTCCTAGGGATAGTGCTAGC	0.045
VP3	CTGACGGCTAGCTCAGTCCTAGGGATAGTGCTAGC	0.065
VP4	TTGATGGCTAGCTCAGTCCTAGGGATTATGCTAGC	0.078
VP5	TTGATGGCTAGCTCAGTCCTAGGTACAGTGCTAGC	0.116
VP6	TTGATGGCTAGCTCAGTCCTAGGTATTGTGCTAGC	0.131
VP7	TTGATGGCTAGCTCAGTCCTAGGTACTATGCTAGC	0.147
VP8	TTGACGGCTAGCTCAGTCCTAGGTACTGTGCTAGC	0.192
VP9	TTGATGGCTAGCTCAGTCCTAGGTACAATGCTAGC	0.235
VP10	TTGATGGCTAGCTCAGTCCTAGGTATAGTGCTAGC	0.265
VP11[c]	TTGACGGCTAGCTCAGTCCTAGGTATTGTGCTAGC	0.297
VP12	TTGATGGCTAGCTCAGTCCTAGGTATAATGCTAGC	0.371
VP13[d]	TTGACGGCTAGCTCAGTCCTAGGTACAGTGCTAGC	0.457
VP14[e]	TTGACAGCTAGCTCAGTCCTAGGTATTGTGCTAGC	0.645
VP15[f]	TTGACAGCTAGCTCAGTCCTAGGTATAATGCTAGC	1.000

[a]Y:C or T, K: G or T, R: A or G, W: A or T.

[b]Based on fluorescence values.

[c]P_{J23118}, [d]P_{J23100}, [e]P_{J23104}, and [f]P_{J23119} promoters are from the Anderson promoter library (http://parts.igem.org/Promoters/Catalog/Anderson).

Supplementary Table 5. Average coefficient weights from the 15 leave-one-out cross-validation results.

-35 motif

Location	A	C	G	T	Random promoter
-35	NA[a]	-0.0783	NA	0.0783	Y (C, T)
-34	NA	NA	NA	NA	T
-33	NA	NA	0.1288	-0.1288	K (G, T)
-32	NA	NA	NA	NA	A
-31	NA	0.1055	NA	-0.1055	Y (C, T)
-30	0.1389	NA	-0.1389	NA	R (A, G)

[a]NA represents an unexplored nucleotide for the random promoter.

-10 motif

Location	A	C	G	T	Random promoter
-12	NA[a]	NA	-0.2251	0.2251	K (G, T)
-11	NA	NA	NA	NA	A
-10	NA	-0.0474	NA	0.0474	Y (C, T)
-9	0.0573	NA	NA	-0.0573	W (A, T)
-8	0.0679	NA	-0.0679	NA	R (A, G)
-7	NA	NA	NA	NA	T

[a]NA represents an unexplored nucleotide for the random promoter.

Supplementary Table 6. Synthetic 5′-UTRs used for translational control

Name	Sequence[a]	ΔG_{UTR}[b]	Relative strength[c]
Template	ACGGAGAWTGCTYAAKSAGTCSTTT	-8.67 ~ 6.48	
UTR1	ACGGAGAATGCTTAATCAGTCGTTT	1.13	0.019
UTR2	ACGGAGATTGCTTAAGCAGTCGTTT	0.28	0.022
UTR3	ACGGAGATTGCTTAATCAGTCCTTT	5.98	0.026
UTR4[d]	ACGGAGATTTGCTTAAGCAGTCGTTT	0.88	0.041
UTR5	ACGGAGAATGCTCAATGAGTCGTTT	-1.22	0.086
UTR6	ACGGAGAATGCTTAATGAGTCGTTT	-2.17	0.139
UTR7	ACGGAGATTGCTTAATGAGTCGTTT	-2.07	0.146
UTR8	ACGGAGAATGCTCAAGGAGTCGTTT	-7.22	0.481
UTR9	ACGGAGATTGCTTAAGGAGTCCTTT	-4.27	0.613
UTR10	ACGGAGAATGCTTAAGGAGTCGTTT	-8.67	0.641
UTR11[d]	ACGGAGAATTGCTCAAGGAGTCGTTT	-7.22	0.700
UTR12	ACGGAGATTGCTTAAGGAGTCGTTT	-8.57	0.702
UTR13[d]	ACGGAGATTGCTTAAGGAGGTCCTTT	-8.18	1.000

[a]W: A or T, Y:C or T, K: G or T, S: C or G.

[b]Calculated value for *sgfp* expression using UTR Library Designer[15].

[c]Based on fluorescence values.

[d]Sequence containing an insertion mutation (the underlined nucleotide).

Supplementary Table 7. Synthetic 5′-UTRs used in this study

Gene	5′-UTR Sequence[a]	ΔG_{UTR}[b]	Predicted expression level[c]
sgfp (P_{lac}, P_{tac}, P_{T7})	AGCGGATAACAATTACGAGGGAAAG	-9.72	1,828,513.08
sgfp (P_{tet}, P_{ara}, P_{J23100})	ATTGCGTGAGAAAGGAGCATCGGGA	-9.94	2,064,841.65
T7 RNAP	GCGGATAACAATTAAGGAGTCCGCG	-1.32	16,662.13
tetR	AGCCGAAGTCTCGAAGGAGCAGGCG	-4.42	94,345.95
gam, exo, beta	GCGGATAACAATTAAGGAGATATGC	-10.67	3,110,669.92
pdc	ATTTGCAGCTAAGAAGGAGATCAAT	-10.22	2,418,513.54
aldB	AAAGTAACCGCGCAAGGAGGGATTC	-10.02	2,162,560.88
budABC	GCGGATAACAATTAAGGAGGTTCCG	-10.67	3,110,669.92
crtEBI	TAACAAAACAAAAGGAGGTAATAGA[d]	-10.74	3,230,036.84
idi	CACGACCATATAAGGAGGATAGAAG[d]	-11.14	4,039,874.16
ispA	CATCAACAAATAAGGAGGAGCTACA[d]	-10.99	3,714,776.60
dxs	CGTCTCCACATAAGGAGCAGTTCAC[d]	-7.04	407,825.27

[a]Underlined sequences are sequences that overlap with the lac operator.

[b,c]Values were calculated using UTR Designer (https://sbi.postech.ac.kr/utr_designer)[16].

[d]These sequences were originally designed in a previous study[17] for lycopene production.

Supplementary Table 8. Comparison of ethanol production with previous studies

Microbial platform	Carbon source	Strategy[a]	Titer[b] (g L^{-1})	Time[b] (h)	Productivity[b] (g L^{-1} h^{-1})	Maximum productivity[b] (g L^{-1} h^{-1})	Yield[b] (g ethanol g^{-1} sugar)	Reference
Sphingomonas sp. A1	Alginate	100-mL medium contained in a 300-mL flask, 150 spm, 30 °C	13.0	77	<0.17	~0.3	~0.28	18
E. coli	Alginate, mannitol, glucose (5:8:1)	100-mL bioreactor, 30 °C	<17	48	<0.35	<0.35	<0.34	19
	Brown macroalgae	1-L bioreactor, 400 rpm, 30 °C	37.8	150	0.25	0.64	0.41	
E. coli	Alginate, mannitol, glucose (5:8:1)	Initial OD_{600} 0.6, 100 mL-medium, 25 °C	22	120	0.18	0.35	0.44	20
S. cerevisiae	DEHU, mannitol (1:2)	High cell density (initial biomass ~7 g), 30-mL medium contained in a 125-mL bottle, 500 rpm, 29 °C	26.2	48	0.55	1.0	~0.41	21
		High cell density (initial biomass ~16 g), 30-mL medium contained in a 125-mL bottle, 500 rpm, 29 °C	36.2	90	0.40	1.9	~0.38	
Vibrio sp. dhg	Alginate, mannitol (1:2)	Initial OD_{600} 0.1, 50-mL medium contained in a 350-mL flask, 200 rpm, 30 °C	25.7	24	1.1	1.8	0.32	This study
	Brown macroalgae	Init OD_{600} 0.05, 1-L medium contained in a 5-L bioreactor, 300 rpm, 30 °C	19.2	24	0.8	1.3	0.32	

[a]spm: stroke per minute, rpm: rotation per minute.

[b]Value was estimated based on given data when it was not explicitly reported.

Supplementary References

1. Zhou, M., Han, F., Li, J. & Zhao, X. Isolation and identification of a novel alginate-degrading bacterium, Ochrobactrum sp. *Songklanakarin J. Sci. Technol.* **30,** 135–140 (2008).
2. Weinstock, M. T., Hesek, E. D., Wilson, C. M. & Gibson, D. G. Vibrio natriegens as a fast-growing host for molecular biology. *Nat. Methods* **13,** 849–851 (2016).
3. Lim, J. H., Seo, S. W., Kim, S. Y. & Jung, G. Y. Refactoring redox cofactor regeneration for high-yield biocatalysis of glucose to butyric acid in Escherichia coli. *Bioresour. Technol.* **135,** 568–573 (2013).
4. Gibson, D. G. *et al.* Enzymatic assembly of DNA molecules up to several hundred kilobases. *Nat. Methods* **6,** 343–345 (2009).
5. Datsenko, K. A. & Wanner, B. L. One-step inactivation of chromosomal genes in Escherichia coli K-12 using PCR products. *Proc. Natl. Acad. Sci. USA* **97,** 6640–6645 (2000).
6. Carver, T., Thomson, N., Bleasby, A., Berriman, M. & Parkhill, J. DNAPlotter: circular and linear interactive genome visualization. *Bioinformatics* **25,** 119–120 (2009).
7. Aziz, R. K. *et al.* The RAST Server: rapid annotations using subsystems technology. *BMC Genomics* **9,** 75 doi: 10.1186/1471-2164-9-75 (2008).
8. Liu, X.-F., Cao, Y., Zhang, H.-L., Chen, Y.-J. & Hu, C.-J. Complete Genome Sequence of Vibrio alginolyticus ATCC 17749T. *Genome Announc.* **3,** e01500-14 doi: 10.1128/genomeA.01500-14 (2015).
9. Frazer, K. A., Pachter, L., Poliakov, A., Rubin, E. M. & Dubchak, I. VISTA: computational tools for comparative genomics. *Nucleic Acids Res.* **32,** W273–W279 (2004).

10. Couronne, O. *et al.* Strategies and tools for whole-genome alignments. *Genome Res.* **13,** 73–80 (2003).

11. Lonetto, M., Gribskov, M. & Gross, C. A. The sigma 70 family: sequence conservation and evolutionary relationships. *J. Bacteriol.* **174,** 3843–3849 (1992).

12. Bervoets, I. *et al.* A sigma factor toolbox for orthogonal gene expression in Escherichia coli. *Nucleic Acids Res.* **46,** 2133–2144 (2018).

13. Sharp, P. M. & Li, W. H. An evolutionary perspective on synonymous codon usage in unicellular organisms. *J. Mol. Evol.* **24,** 28–38 (1986).

14. Hoffart, E. *et al.* High substrate uptake rates empower Vibrio natriegens as production host for industrial biotechnology. *Appl. Environ. Microbiol.* **83,** e01614–e01617 (2017).

15. Seo, S. W. *et al.* Predictive combinatorial design of mRNA translation initiation regions for systematic optimization of gene expression levels. *Sci. Rep.* **4,** 4515 doi: 10.1038/srep04515 (2014).

16. Seo, S. W. *et al.* Predictive design of mRNA translation initiation region to control prokaryotic translation efficiency. *Metab. Eng.* **15,** 67–74 (2013).

17. Jung, J. *et al.* Precise precursor rebalancing for isoprenoids production by fine control of gapA expression in Escherichia coli. *Metab. Eng.* **38,** 401–408 (2016).

18. Takeda, H., Yoneyama, F., Kawai, S., Hashimoto, W. & Murata, K. Bioethanol production from marine biomass alginate by metabolically engineered bacteria. *Energy Environ. Sci.* **4,** 2575-2581 (2011).

19. Wargacki, A. J. *et al.* An engineered microbial platform for direct biofuel production from brown macroalgae. *Science* **335,** 308–313 (2012).

20. Santos, C. N. S., Regitsky, D. D. & Yoshikuni, Y. Implementation of stable and complex biological systems through recombinase-assisted genome engineering. *Nat. Commun.* **4,** 2503 doi: 10.1038/ncomms3503 (2013).

21. Enquist-Newman, M. *et al.* Efficient ethanol production from brown macroalgae sugars by a synthetic yeast platform. *Nature* **505,** 239–243 (2014).
