# Supplementary material for: Vibrio sp. dhg as a platform for the biorefinery of brown macroalgae
Source: Nat Commun. 2019 Jun 6;10:2486. doi: 10.1038/s41467-019-10371-1 (PMC6554313; doi:10.1038/s41467-019-10371-1)
Supplement: Supplementary file 4 — Description of Additional Supplementary Files [file 41467_2019_10371_MOESM4_ESM.docx]

**Description of Additional Supplementary Files**

File Name: Supplementary Data 1

Description: List of strains and plasmids used in this study

File Name: Supplementary Data 2

Description: List of primers used in this study

File Name: Supplementary Data 3

Description: Sequence of the 16S rDNA of *Vibrio* sp. dhg

File Name: Supplementary Data 4

Description: List of catabolic enzymes for sugar utilization in *Vibrio* sp. dhg
