# Supplementary material for: Vibrio sp. dhg as a platform for the biorefinery of brown macroalgae
Source: Nat Commun. 2019 Jun 6;10:2486. doi: 10.1038/s41467-019-10371-1 (PMC6554313; doi:10.1038/s41467-019-10371-1)
Supplement: Supplementary file 2 — Peer Review File [file 41467_2019_10371_MOESM2_ESM.pdf]

Reviewers' comments:

Reviewer #1 (Remarks to the Author):

This a generally well written, original and interesting article on the use of Vibrio sp. Dhg (a Gram-negative halophilic bacterium) to utilise macroalgal sugars, such as alginate, which are generally resistant to hydrolysis by conventional microbial platforms. The paper gives genomic information and details of its genetic modification to improve the yield demonstrated of ethanol, 2,3-butanediol, and lycopene from a sugar mixture typical of brown macroalgae. The paper appears suitable for publication in Nature Communications and should be of interest to algal biotechnology research community.

The paper requires only minor corrections:
Define μ on first use.
Correct grammar throughout.
There is considerable use of personal pronouns.
The title, "Vibrio sp. dhg as a new powerful platform for the biorefinery of brown macroalgae" may overstate the current position of the research.
Units are generally much clearer for complex units if mg L-1 notation, not mg/L is used. Order for complex units is [number - unit - identifier] e.g. 10 mg VS L-1 or 0.4 L CH4 g-1 VS.

Reviewer #2 (Remarks to the Author):

This article provides a powerful and an alternative system to utilize alginate for biorefinery; developing a new bacterial-host and fine genetic tools. This article would be acceptable to specialized journal of high quality. However, this is the Nature Communications and my decision depends on the responses of authors to my concerns.

1: Authors isolated a marine bacterium. In general, bacteria are sensitive to fermentation-inhibitors and sever conditions, such as salts, ethanol, organic acids, organic solvents, etc. This would be one of drawbacks when compared with e.g. yeast. Thus, I request that authors should present how Vibrio sp. dhg strain shows tolerance to these stresses and should compare them with those of E. coli and S. cerevisiae, and if possible, other representative hosts. These data will help readers to see how the dhg strain is good or bad.

2: p. 8 lines 183- p. 9 lines 185: Authors used a mixture of alginate and mannitol in a 1:2 ratio since this is "the natural sugar composition of brown macroalgae". However, this is never the natural composition because mannitol content in brawn macroalgae dramatically varies during a year although alginate content is relatively constant. Moreover, authors described that "As these two sugars have different oxidation states (p. 5, lines 108-109)". Thus authors should also use other compositions of the alginate and mannitol at 1:0, 0:1, and 1:1 for the productions of biochemicals. These data would be very valuable to understand the metabolisms of these sugars with different oxidation states. Production of biochemicals from alginate, not mannitol, using bacterial system would be significant. Readers would want to see how alginate contribute to the production and how mannitol helps it.

Furthermore, productions from extracts of brown macroalgae are very important. Do the dhg strain produce biochemicals or fuels from extracts of brown macroalgae ? I wonder if the strain show sensitivity or tolerance to the algal-derived components, which is also related with my concern above.

3: p. 9, line 206: Authors described “under similar conditions ref 5, 19 ,20”. However, they are never similar conditions. Authors should describe the details of the conditions conducted and the results obtained in the ref 5, 19, 20.

4: Supplementary Note 3: I wonder if these reactions make sense because authors cultivate the dhg strain in highly aerobic conditions.

5: Minor concern, Supplementary Note 3: 2 Pyruvate + 2 NADH + 2H+ = 2 Ethanol + 2 NAD+ + 2 CO2 would be correct. Others also should be corrected. NAD+, not NAD, is correct.

6: Minor concern, Supplementary Note 1. Please specify the pH of the medium.

Reviewer #3 (Remarks to the Author):

Comments on manuscript Nature Communications NCOMMS-18-32598 by Lim et al.

Lim et al. isolated an alginate metabolizing organism from seaweed sludge. Genome sequencing identified the isolate to be closely related to the fast-growing Vibrio natriegens which is not able to metabolize alginate. The authors designated the isolate as Vibrio sp. dhg and show that this strain shows high growth rates on alginate and mannitol and is able to consume both carbon sources in parallel. The authors established basic genetic engineering tools and engineered Vibrio sp. dhg for the production of ethanol, 2,3-butanediol and lycopene.

General comments / Major points
- The authors sequenced the genome and identified a plasmid (143 kb) which is not present in V. natriegens (Supp. Fig. 1). However, other closely related alginate metabolizing Vibrios such as V. alginolyticus are pathogens. Surprisingly, the authors do not give any information about the presence or absence of any factors indicating pathogenicity which might be located on the plasmid or on the genome. This information is essential and evidence of pathogenicity factors will render industrial application of Vibrio sp. dhg almost impossible. Therefore, this point has to be clarified as Weinstock et al. 2016 did for V. natriegens.

- In l. 69-71 (Fig. 1b and c) the authors state that the growth rates and sugar uptake rates (qS) are substantially higher compared to E. coli. The given E. coli values (Fig. 1b and c) are rather low. Varma and Palsson (1994, Appl Environ Microbiol 60:3724-3731.) gives growth rates for E. coli during growth on glucose of 0.68 with corresponding qS values of 1.9 g g-1 h-1 under aerobic conditions. Under anaerobic conditions the qS for E. coli is even higher with 3.3 g g-1 h-1 (Varma and Palsson, 1994). Therefore, especially the given qS for E. coli in Fig. 1c is by far too low and the comparison does not provide a correct picture for the reader. Moreover, growth rates and qS values of V. natriegens are significantly higher compared to Vibrio sp. dhg and E. coli (with the exception for alginate) (Hoffart et al. 2017). Therefore, the authors should compare growth rate and qS of Vibrio sp. dhg with values of V. natriegens. The growth rate of Vibrio sp. dhg on alginate should also be discussed with other relevant alginate metabolizing microorganism. These experiments have been performed in shake flasks which represents, regarding oxygen availability, a rather undefined environment. Since qS values might differ significantly between aerobic and aerobic conditions the authors might perform the experiments at least for one substrate under defined bioreactor conditions.

- In Fig. 1b and c: During aerobic growth on glucose most bacteria show a biomass yield of about 40 – 50%. For E. coli and V. natriegens under aerobic conditions about 40% of the carbon will be found in biomass, 10% in acetate and 50% in CO2. Under anaerobic conditions the biomass yield will decrease

to about 20% for both organism. In Fig. 1 b and c a growth rate of about 1.05 h-1 and a qS of about 1 g g-1 h-1 can be found on glucose. With both values one can calculate a biomass yield of about 1 g

g-1, which is hardly possible. Unfortunately, it is therefore questionable if the given data is reliable.
- Fig. 3: As stated above the culture conditions are not well defined. Why do the strains stop growing although plenty of sugar is present (Fig. 3 b and d)? Is it any limitation or does product inhibition already occur? Please clarify.

Minor points
- L. 28: Replace develop by establish
- L. 31-33: This statement is very optimistic since an industrial relevant proof of concept is not given
- L. 39: The authors should compare alginate with lignocellulosic feedstocks which does also not compete as food resource. For this reason macroalgae is not a “more attractive (l. 41)” feedstock.
L.42: the C-content of lignocellulosic feedstocks is about 50% and therefore significant higher compared to macroalgae.
- L.248-249: The statement is too general. This holds only true for alginate.
- Fig. 8: Please provide information about the genotype of each strain

Responses to reviewers' comment

We greatly appreciate the editor and reviewers' effort in reviewing this manuscript. The critical comments from the reviewers were fruitful to improve our manuscript. We have addressed all issues and included responses. We believe that the revised version will meet the requirements of *Nature Communications*.

Comments from reviewer #1

This a generally well written, original and interesting article on the use of Vibrio sp. Dhg (a Gram-negative halophilic bacterium) to utilise macroalgal sugars, such as alginate, which are generally resistant to hydrolysis by conventional microbial platforms. The paper gives genomic information and details of its genetic modification to improve the yield demonstrated of ethanol, 2,3-butanediol, and lycopene from a sugar mixture typical of brown macroalgae. The paper appears suitable for publication in Nature Communications and should be of interest to algal biotechnology research community. The paper requires only minor corrections:

(Response) We thank the reviewer for the positive comments and provide detailed response to each comment below.

Comment 1. Define μ on first use.

(Response) According to the reviewer's comment, μ was defined before its use (line 68 of page 4). In addition, calculation methods for physiological parameters were explicitly detailed in Methods section.

Comment 2. Correct grammar throughout.

(Response) According to the reviewer's comment, we thoroughly reviewed and corrected grammatical errors in the revised manuscript.

Comment 3. There is considerable use of personal pronouns.

(Response) According to the reviewer's comment, the manuscript was revised to avoid unnecessary uses of personal pronoun.

Comment 4. The title, "Vibrio sp. dhg as a new powerful platform for the biorefinery of brown macroalgae" may overstate the current position of the research.

(Response) To reflect the reviewer's comment, we changed our title to "*Vibrio* sp. dhg as a promising platform for the biorefinery of brown macroalgae"

Comment 5. Units are generally much clearer for complex units if mg L-1 notation, not mg/L is used. Order for complex units is [number - unit - identifier] e.g. 10 mg VS L-1 or 0.4 L CH4 g-1 VS.

(Response) According to the reviewer's comment, units were changed accordingly.

Comments from reviewer #2

This article provides a powerful and an alternative system to utilize alginate for biorefinery; developing a new bacterial-host and fine genetic tools. This article would be acceptable to specialized journal of high quality. However, this is the Nature Communications and my decision depends on the responses of authors to my concerns.

(Response) We thank the reviewer for the positive comments and provide detailed response to each comment below.

Comment 1. Authors isolated a marine bacterium. In general, bacteria are sensitive to fermentation-inhibitors and sever conditions, such as salts, ethanol, organic acids, organic solvents, etc. This would be one of drawbacks when compared with e.g. yeast. Thus, I request that authors should present how *Vibrio* sp. dhg strain shows tolerance to these stresses and should compare them with those of *E. coli* and *S. cerevisiae*, and if possible, other representative hosts. These data will help readers to see how the dhg strain is good or bad.

(Response) According to the comment, we cultivated *Vibrio* sp. dhg and other representative hosts (*Escherichia coli*, *Corynebacterium glutamicum*, and *Saccharomyces cerevisiae*) in the presence of various chemicals (salt-NaCl, ethanol, lactic acid, and 2,3-BDO) with different concentrations. As shown in Fig. S1 of the revised manuscript, *Vibrio* sp. dhg can grow even in a high concentration of salt (100 g L^{-1} of NaCl) while the other industrial hosts showed severely reduced growth rates. In the case of ethanol, this microorganism was similar to in *C. glutamicum* and maintained its growth with 50 g L^{-1} of ethanol in the medium. For 2,3-BDO, it was similar to *E. coli* but more sensitive than *C. glutamicum* and *S. cerevisiae*; the growth was inhibited by 50 g L^{-1} of 2,3-BDO. Finally, with lactate, its tolerance was slightly less than the tolerance of *S.*

cerevisiae which showed the best growth in the presence of 50 g L^{-1} of lactate. Overall, although its tolerance was not much superior to either *C. glutamicum* or *S. cerevisiae*, it was comparable to *E. coli*, suggesting this microorganism can be used as a host for biochemical production. Furthermore, tolerance engineering that has been made for the conventional microorganisms could be helpful to improve the tolerance for maximizing its potential as a platform strain. To include the data, we revised the manuscript as follow:

"In addition, this microorganism can grow even in a high concentration of salt (100 g L^{-1} of sodium chloride) while other industrial hosts (E. coli, Corynebacterium glutamicum, Saccharomyces cerevisiae, Supplementary Data 1, and Supplementary Fig. 1a) showed severely reduced growth rates. Tolerance to other representative bioproducts (ethanol, 2,3-butanediol (2,3-BDO), and lactate) was also investigated (Supplementary Fig. 1b-d). In the case of ethanol, this microorganism was similar to C. glutamicum and maintained its growth with 50 g L^{-1} of ethanol in the medium. For 2,3-BDO, it was similar to E. coli but more sensitive than C. glutamicum and S. cerevisiae; the growth was inhibited by 50 g L^{-1} of 2,3-BDO. Finally, with lactate, its tolerance was slightly less than the tolerance of S. cerevisiae which showed the best growth in the presence of 50 g L^{-1} of lactate. Overall, although its tolerance was not much superior to either C. glutamicum or S. cerevisiae, it was comparable to E. coli, suggesting this microorganism can be used as a host for biochemical production." (Line 73-84 of page 4 in the revised version)

"Further studies aimed at unveiling the characteristics (e.g., rapid growth, halophilicity) and improving its tolerance[30,31] *of Vibrio sp. dhg would be valuable for maximizing its potential as a microbial platform*[32]*"* (Line 305-307 of page 13 in the revised version)

Comment 2. p. 8 lines 183- p. 9 lines 185: Authors used a mixture of alginate and mannitol in a 1:2 ratio since this is “the natural sugar composition of brown macroalgae”. However, this is never the natural composition because mannitol content in brawn macroalgae dramatically varies during a year although alginate content is relatively constant. Moreover, authors described that “As these two sugars have different oxidation states (p. 5, lines 108-109)”. Thus authors should also use other compositions of the alginate and mannitol at 1:0, 0:1, and 1:1 for the productions of biochemicals. These data would be very valuable to understand the metabolisms of these sugars with different oxidation states. Production of biochemicals from alginate, not mannitol, using bacterial system would be significant. Readers would want to see how alginate contribute to the production and how mannitol helps it.

(Response) We strongly agree that composition of natural biomass can be varied depending on seasonal and geographical changes, thus related study should be valuable to develop brown macroalgae refinery process. To investigate the effect, we cultivated the VDHG411 strain that was engineered for ethanol production with different ratios of alginate and mannitol (Fig. S12 in the revised version). Although the VDHG411 strain was able to produce ethanol regardless of medium composition, the titer as well as sugar consumption was significantly affected by the composition. As we expected and discussed in Supplementary Note 3, best ethanol production was achieved when 1:2 mixture of alginate and mannitol was provided as carbon sources. We believe that further engineering based on our genetic engineering toolbox can improve the performance in future studies. To reflect this issue, we revised the manuscript to explain this issue as follow:

“For the realization of the brown macroalgae biorefinery, there could still be many hurdles. As mentioned, one challenge is a potential fluctuation of bioprocess efficiency due to

seasonal and geographical differences in biomass sugar composition. Indeed, we observed that the ratio of alginate and mannitol, which have different oxidation states, hugely affected ethanol production (up to 4.5-fold) using our engineered strain (Supplementary Fig. 12 and Supplementary Note 3). To address this issue, microorganisms should be engineered for dynamic response to intracellular redox state for robust biochemical production with advantages of recent advances in synthetic biology[26-29]." (Line 297-304 of page 13 in the revised version)

Comment 2-2. Furthermore, productions from extracts of brown macroalgae are very important. Do the dhg strain produce biochemicals or fuels from extracts of brown macroalgae? I wonder if the strain show sensitivity or tolerance to the algal-derived components, which is also related with my concern above.

(Response) According to the reviewer's comment, we cultivated our engineered strain with providing brown macroalgae as a feedstock (Fig. 4 in the revised version). Surprisingly, even without any enzymatic pre-treatment or hydrolysis, we were able to produce 19.2 g L^{-1} during 24 h, similar performance from mimetic sugar mixture fermentation. We added the data and revised the manuscript as follow:

"*Finally, ethanol production was attempted using brown macroalgae (Fig. 4). The sugar content of used Kelp powder was quantified to 0.16 g alginate and 0.3 g mannitol per g powder (see Methods), consistent with the composition of the mimetic sugar mixture. Therefore, the Kelp powder can be directly used as a feedstock. When we cultivated the VDHG411 strain in a bioreactor containing 1-L medium, total 120 g L^{-1} of the Kelp powder was provided during 24 h. Subsequently, even without any enzymatic pre-treatment or hydrolysis, 19.2 g L^{-1} of ethanol (2.4 % v/v) was successfully produced with a similar yield (63% of theoretical maximum) compared to*

the yield from mimetic sugar fermentation. Although the productivity (0.8 g L^{-1} h^{-1}) was reduced, suggesting requirement of further optimization including genetic engineering and parameter study, the obtained result clearly indicates that Vibrio sp. dhg is also capable to produce a biochemical directly from brown macroalgae." (Line 266-278 of page 12 in the revised version)

"For ethanol production from brown macroalgae, a powder of kelp (60 mesh) harvested in Korea was purchased from a local market. Its sugar composition was quantified using 10 g L^{-1} of the kelp solution with water. Culture was conducted in the bioreactor containing the 1-L buffered medium with 10 g L^{-1} of alginate and mannitol (1:2) as initial carbon sources. Especially, phosphate buffer concentration was adjusted to 20 mM and 5 g L^{-1} of yeast extract were additionally provided. Sterile air at 2 L min^{-1} was provided during initial 6 h. Then, the cells were cultured anaerobically and 20 g L^{-1} of the kelp powder was added with a 3-h interval. The culture was continuously stirred at 300 rpm." (Line 490-497 of page 22 in the revised version)

Comment 3. p. 9, line 206: Authors described "under similar conditions ref 5, 19 ,20". However, they are never similar conditions. Authors should describe the details of the conditions conducted and the results obtained in the ref 5, 19, 20.

(Response) To reflect the reviewer's comment, we additionally included Supplementary Table 8 in the revised version to compare culture conditions and obtained results. Our strain showed higher productivity compared to that of microbial platforms.

Comment 4. Supplementary Note 3: I wonder if these reactions make sense because authors cultivate the dhg strain in highly aerobic conditions.

(Response) Aeration directly affects the activity of the electron transport chain which uses oxygen as the final electron acceptor. Therefore, aeration should be carefully accounted when ATP generation from oxidation of reducing equivalents (e.g., NADH) is critical for biochemical production. However, reactions in Supplementary Note 3 does not require generation of ATP from NADH. Therefore, our discussion can explain both aerobic and oxygen-limited conditions.

Comment 5: Minor concern, Supplementary Note 3: 2 Pyruvate + 2 NADH + 2H+ = 2 Ethanol + 2 NAD+ + 2 CO2 would be correct. Others also should be corrected. NAD+, not NAD, is correct.

(Response) We apologize for the mistake. The errors were corrected in the revised version.

Comment 6. Minor concern, Supplementary Note 1. Please specify the pH of the medium.

(Response) According to the reviewer's comment, we revised the Supplementary Note 1.

Comments from reviewer #3

Comments on manuscript Nature Communications NCOMMS-18-32598 by Lim et al.

Lim et al. isolated an alginate metabolizing organism from seaweed sludge. Genome sequencing identified the isolate to be closely related to the fast-growing Vibrio natriegens which is not able to metabolize alginate. The authors designated the isolate as Vibrio sp. dhg and show that this strain shows high growth rates on alginate and mannitol and is able to consume both carbon sources in parallel. The authors established basic genetic engineering tools and engineered Vibrio sp. dhg for the production of ethanol, 2,3-butanediol and lycopene.

(Response) We thank the reviewer's effort in reviewing this manuscript and provide detailed response to each comment below.

Comment 1. The authors sequenced the genome and identified a plasmid (143 kb) which is not present in V. natriegens (Supp. Fig. 1). However, other closely related alginate metabolizing Vibrios such as V. alginolyticus are pathogens. Surprisingly, the authors do not give any information about the presence or absence of any factors indicating pathogenicity which might be located on the plasmid or on the genome. This information is essential and evidence of pathogenicity factors will render industrial application of Vibrio sp. dhg almost impossible. Therefore, this point has to be clarified as Weinstock et al. 2016 did for V. natriegens.

(Response) We appreciate your excellent comment. When we analyzed the genome of *Vibrio* sp. dhg with following the method (Weinstock et al. 2016), we were not able to find major toxins. However, one barely expressed open reading frame (fragments per kilobase per million (FPKM) <1 based on RNA-seq analysis) showed a marginal identity (24%, Supplementary Fig. 13a) when compared its amino acid sequence with an auxiliary toxin (Zot, zona occludens toxin).

Although it has a low sequence identity and *Vibrio* sp. dhg strain does not have two major toxins (CtxA and CtxB) concurrently found with Zot, we tested that the potential toxin can be deleted to minimize the risk. Finally, we confirmed that this gene can be deleted and does not affect the growth of the strain (Supplementary Fig. 11c and d). To inform this potential virulence of environmental isolates to readers, we included the analysis result and added a paragraph in discussion section.

"*One risk in exploiting environmental isolates as a host is their potential virulence. Our genome analysis revealed that Vibrio sp. dhg strain does not have major toxins found from pathogenic Vibrio strains*[10] *(see Methods). However, one barely expressed open reading frame (fragments per kilobase per million (FPKM) <1 based on RNA-seq analysis) showed a marginal identity (24%, Supplementary Fig. 11a and b) when compared with its amino acid sequence with an auxiliary toxin (Zot, zona occludens toxin). Although it has a low sequence identity (less than 30%*[24]*) and Vibrio sp. dhg strain does not have two major toxins (CtxA and CtxB) concurrently found with Zot*[25]*, we tested that the potential toxin can be deleted to minimize the risk. We confirmed that this gene can be deleted and does not affect the growth of the strain (Supplementary Fig. 11c and d).*" (Line 287-296 of page 13 in the revised version)

"*Potential pathogenicity of Vibrio. sp dhg was evaluated by searching known Vibrio toxins (Ace, CT, MARTX, TDH, TRH, VCC, and Zot) using Basic Local Alignment Search Tool (BLAST, https://blast.ncbi.nlm.nih.gov/Blast.cgi?PROGRAM=tblastn)*[10,42,43]*. Amino acid sequence of the toxins and genome sequence of Vibrio sp. dhg were used as inputs.*" (Line 517-521 of page 23 in the revised version)

Reference:

Weinstock, M. T., Hesek, E. D., Wilson, C. M. & Gibson, D. G. Vibrio natriegens as a fast-growing host for molecular biology. Nat. Methods 13, 849–851 (2016).

Comment 2. In l. 69-71 (Fig. 1b and c) the authors state that the growth rates and sugar uptake rates (qS) are substantially higher compared to E. coli. The given E. coli values (Fig. 1b and c) are rather low. Varma and Palsson (1994, Appl Environ Microbiol 60:3724-3731.) gives growth rates for E. coli during growth on glucose of 0.68 with corresponding qS values of 1.9 g g-1 h-1 under aerobic conditions. Under anaerobic conditions the qS for E. coli is even higher with 3.3 g g-1 h-1 (Varma and Palsson, 1994). Therefore, especially the given qS for E. coli in Fig. 1c is by far too low and the comparison does not provide a correct picture for the reader.

(Response) We apologize for the miscalculation of the specific sugar uptake rates. Those values were carefully re-calculated with equations that used in the previous studies (Varma and Palsson, 1994; Hoffart et al, 2017, also mentioned in Methods section). With our revised data, growth rate (0.60 h^{-1}) and sugar uptake rate (2.0 g g^{-1} DCW h^{-1}) of *E. coli* W3110 at 37 ^{o}C were similar to the previously reported growth rate (0.68 h^{-1}) and sugar uptake rate (1.9 g g^{-1} DCW h^{-1}) at 38 ^{o}C (Varma and Palsson, 1994). As shown in the revised Fig. 1, growth rates and sugar uptake rates of *Vibrio* sp. dhg are still significantly higher or comparable when compared with the values of *E. coli*.

Reference:

Varma, A. & Palsson, B. O. Stoichiometric flux balance models quantitatively predict growth and metabolic by-product secretion in wild-type Escherichia coli W3110. Appl. Environ. Microbiol. 60, 3724–3731 (1994).

Hoffart, E. et al. High substrate uptake rates empower Vibrio natriegens as production host for industrial biotechnology. Appl. Environ. Microbiol. 83, e01614–e01617 (2017).

Comment 2-2. Moreover, growth rates and qS values of *V. natriegens* are significantly higher compared to *Vibrio* sp. dhg and *E. coli* (with the exception for alginate) (Hoffart et al. 2017). Therefore, the authors should compare growth rate and qS of *Vibrio* sp. dhg with values of *V. natriegens*. The growth rate of *Vibrio* sp. dhg on alginate should also be discussed with other relevant alginate metabolizing microorganism.

(Response) According to the reviewer's comment, we compared *V. natriegens* and *Vibrio* sp. dhg (Supplementary Table 3 in the revised version). We observed that both growth rate and sugar uptake rate of *Vibrio* sp. dhg were slightly lower than the rates of *Vibrio natriegens*, probably due to a variation in more than 5% of their genomes. However, the metabolic activity of *Vibrio* sp. dhg is still much higher metabolic activity than *E. coli*. In addition, we also mentioned that growth rate (0.98 h^{-1}) of *Vibrio* sp. dhg is higher than previously reported growth rates (0.2-0.8 h^{-1}) (Hehemann et al., 2016) of other microorganisms during alginate utilization. Manuscript was revised to include the comparison accordingly.

"Although several Vibrio species are known to use alginate (e.g., V. alginolyticus and V. splendidus 12B01), its growth rate is much higher than reported values of the known microorganisms (0.2-0.8 h^{-1})[7]. Moreover, the genome context and phenotypic characteristics of our microorganism were closer to those of V. natriegens ATCC14048 (Supplementary Figs. 3

and 4). However, detailed physiological comparison (Supplementary Table 3) revealed that there is a difference between two microorganisms, probably due to a variation in more than 5% of their genomes. Particularly, V. natriegens does not possess the machinery needed to metabolize alginate. Therefore, we named our novel strain Vibrio sp. dhg and further investigated its potential as a platform for biorefinery from brown macroalgae." (Line 92-101 of page 5 in the revised version)

Reference:

Hehemann, J.-H. et al. Adaptive radiation by waves of gene transfer leads to fine-scale resource partitioning in marine microbes. Nat. Commun. 7, 12860 (2016).

Comment 2-3. These experiments have been performed in shake flasks which represents, regarding oxygen availability, a rather undefined environment. Since qS values might differ significantly between aerobic and aerobic conditions the authors might perform the experiments at least for one substrate under defined bioreactor conditions.

(Response) According to the reviewer's comment, we measured qS values of *Vibrio* sp. dhg in both aerobic and anaerobic reactor conditions with glucose as a carbon source (Supplementary Table 3 in the revised version). We observed a similar trend in the growth rate and the sugar uptake rate.

Comment 3. In Fig. 1b and c: During aerobic growth on glucose most bacteria show a biomass yield of about 40 – 50%. For E. coli and V. natriegens under aerobic conditions about 40% of the carbon will be found in biomass, 10% in acetate and 50% in CO2. Under anaerobic conditions

the biomass yield will decrease to about 20% for both organism. In Fig. 1 b and c a growth rate of about 1.05 h-1 and a qS of about 1 g g-1 h-1 can be found on glucose. With both values one can calculate a biomass yield of about 1 g g-1, which is hardly possible. Unfortunately, it is therefore questionable if the given data is reliable.

(Response) We also apologize for the confusion. With the revised data (Supplementary Table 3 in the revised version), we re-calculated the biomass yield of *Vibrio* sp. dhg. At 30 ^{o}C, we found that *Vibrio* sp. dhg shows 41% and 20% in aerobic and anaerobic condition, respectively. Those values are consistent with values of most bacteria including *V. natriegens*.

Comment 4. Fig. 3: As stated above the culture conditions are not well defined. Why do the strains stop growing although plenty of sugar is present (Fig. 3 b and d)? Is it any limitation or does product inhibition already occur? Please clarify.

(Response) For biochemical production, we used a buffered medium containing 5 g L^{-1} of $(NH_4)_2SO_4$ as a nitrogen source. Because it is known that nitrogen consists 14% of dry cell weight (Pauline 1995), less than 8 g DCW L^{-1} (equivalent to OD_{600} <30) of biomass is obtainable from 5 g L^{-1} of $(NH_4)_2SO_4$. In both ethanol and 2,3-BDO production cases, cells grew rapidly and reached OD_{600} 25~30 after 12 h. Therefore, after 12 h, cells might not be able to grow further due to nitrogen-limitation rather than product inhibition as *Vibrio* sp. dhg seems to be tolerant until 20 g L^{-1} of either ethanol and 2,3-BDO (Supplementary Figure 1). Additional nitrogen source was not supplemented to avoid too much accumulation of biomass, which potentially lowers the production yields.

Reference:

Pauline M. Doran. Bioprocess Engineering Principles Ch. 4 (Elsevier, 1995).

Comment 5. L. 28: Replace develop by establish

(Response) According to the reviewer's comment, we revised the manuscript.

Comment 6. L. 31-33: This statement is very optimistic since an industrial relevant proof of concept is not given

(Response) According to the reviewer's comment, we revised the manuscript as follow:

"Collectively, Vibrio sp. dhg can be a promising platform for the efficient conversion of brown macroalgae sugars into diverse value-added biochemicals." (Line 33-34 of page 2 in the revised version)

Comment 7. L. 39: The authors should compare alginate with lignocellulosic feedstocks which does also not compete as food resource. For this reason macroalgae is not a "more attractive (l. 41)" feedstock.

(Response) To reflect the reviewer's comment, we revised the manuscript as follow:

"In this respect, brown macroalgae has been suggested as an alternative feedstock. Brown macroalgae is hugely abundant and has a high carbohydrate content (35-60% of dry weight)[2-4]. They can grow much faster than lignocellulosic biomasses and only require sunlight and seawater[5]" (Line 40-43 of page 3 in the revised version)

Comment 8. L.42: the C-content of lignocellulosic feedstocks is about 50% and therefore significant higher compared to macroalgae.

(Response) According to our analysis of Kelp composition, its sugar content was estimated to be 46%. In addition, we found a reference (Holdt & Kraan, 2011) which reports sugar compositions (35-60% of dry weight) of various macroalgae. To reflect this issue, we revised the manuscript as follow:

"In this respect, brown macroalgae has been suggested as an alternative feedstock. Brown macroalgae is hugely abundant and has a high carbohydrate content (35-60% of dry weight)[2-4]. They can grow much faster than lignocellulosic biomasses and only require sunlight and seawater[5]" (Line 40-43 of page 3 in the revised version)

"Sugar content of used Kelp powder was quantified to 0.16 g alginate and 0.3 g mannitol per g powder (see Methods), consistent with the composition of the mimetic sugar mixture." (Line 267-269 of page 12 in the revised version)

Reference:

Holdt, S. L. & Kraan, S. Bioactive compounds in seaweed: functional food applications and legislation. J Appl Phycol 23, 543–597 (2011).

Comment 9. L.248-249: The statement is too general. This holds only true for alginate.

(Response) To reflect the reviewer's comment, we revised the manuscript as follow:

"Moreover, its outstanding growth rate and metabolic activity over conventional platforms allow envisioning accelerated biochemical production from brown macroalgae." (Line 283-285 of page 12-13 in the revised version)

Comment 10. Fig. 8: Please provide information about the genotype of each strain

(Response) To reflect the reviewer's comment, we provided the genotype of each strain in the captions of Supplementary Fig. 9 and 10.

Additional modifications in the manuscript during the revision:

- S.H.W. is a new co-first author due to his substantial contribution during revision. Author contributions section was accordingly revised.
- Subheadings were added in the text.
- Supplementary Table 1, 2, and 6 in the original version were changed to Supplementary Data 1,2,3 due to their large sizes.
- We combined Supplementary Fig. 1 and Supplementary Table 3 of the original version into Supplementary Fig. 2 for better presentation.
- We revised Supplementary Fig. 6 to include detailed alignment results.
- We acknowledged H.H.J. for his technical supporting and Creative-Pioneering Researchers Program through Seoul National University (SNU) for its financial support in Acknowledgments section.

REVIEWERS' COMMENTS:

Reviewer #1: Unavailable.

Reviewer #2 (Remarks to the Author):

My concerns are fully satisfied and I suggest publication of this revised article in Nature Communications.

Reviewer #3 (Remarks to the Author):

All points raised by the reviewer have been adreesed adequately.
